# The Effectiveness of Early Intervention Programs for Children Aged 0–3: A Systematic Review and Meta-Analysis

**DOI:** 10.3390/children13101324

**Published:** 2026-09-29

**Authors:** Min Qiu, Shuhua Zhang, Weiyan Liao, Hui Li

**Affiliations:** Faculty of Education and Human Development, The Education University of Hong Kong, Hong Kong SAR, China

**Keywords:** early intervention, infants and toddlers, neurodevelopment, meta-analysis, physical growth

## Abstract

**Highlights:**

**What are the main findings?**

- Early intervention for infants aged 0–3 significantly improves physical growth and clinic-based motor skills, though overall effects on motor development remain statistically non-significant due to outcome variation.
- Exploratory subgroup analyses suggested larger pooled effects among professionally led interventions and interventions lasting less than six months, although evidence certainty remained low to very low.

**What are the implications of the main findings?**

- Exploratory subgroup analyses suggested larger pooled effects for professionally guided interventions; however, evidence certainty remained low to very low, and no causal inference can be drawn.
- Interventions targeting high-risk populations, such as preterm infants, should tailor strategies to specific developmental domains rather than relying on uniform, broad-spectrum models.

**Abstract:**

Background: Existing syntheses on infant early interventions focus on single-risk cohorts and overlook implementation moderators. To address these gaps, this systematic review and meta-analysis adopts a transdiagnostic approach to evaluate interventions across diverse risk profiles and identify predictive intervention characteristics, including setting, provider, and duration. Methods: PubMed, Web of Science, Scopus, CINAHL, and PsycINFO were searched up to February 2026 for peer-reviewed English randomized controlled trials (RCTs), cluster RCTs, or quasi-RCTs evaluating early interventions for children aged 0–3, control groups, and extractable developmental data. Study quality was assessed using the Cochrane Risk of Bias 2 tool. Standardized mean differences (SMDs) were pooled using a random-effects model, with subgroup analyses investigating the influence of intervention duration, setting, provider, and infant risk factors (PROSPERO: CRD420261436569). Results: Across 17 studies (*n* = 1987), early intervention did not have a statistically significant overall benefit for motor development (SMD = 0.49, *p* = 0.12). However, exploratory subgroup analyses suggested larger pooled motor effects among professionally delivered and clinic-based interventions (SMD = 1.16), while exploratory language benefits were observed among interventions lasting less than six months (SMD = 1.52). For preterm infants, targeted interventions suggested initial promise for physical growth (SMD = 0.55) but not motor function (SMD = 0.69, *p* = 0.60). Conclusions: Intervention effectiveness depends on delivery format, setting, and population. Exploratory analyses suggested that intervention characteristics such as provider type and duration may contribute to outcome variability across domains. Despite small trial sample sizes and high statistical heterogeneity, these overall estimates should be interpreted as descriptive benchmarks rather than uniform treatment effects; interpretation therefore focuses on subgroup findings.

## 1. Introduction

The first three years of life represent a period of unparalleled neural plasticity and organizational development. During this critical window, over 80% of the brain’s structural architecture is established, forming the foundation for all subsequent cognitive, motor, and social–emotional competencies [1]. This rapid development is not merely a passive, predetermined process. Influential theories like neuroconstructivism posit that the brain is actively shaped through dynamic, bidirectional interactions between a child’s genetic predispositions and their environmental inputs [2]. This perspective provides a powerful rationale for early intervention: targeted, high-quality experiences delivered during this malleable period have a heightened potential to alter developmental trajectories and mitigate the effects of early adversity.

The importance of the child’s environment is further underscored by both relational and systemic theories. Attachment theory, for instance, highlights the primacy of secure caregiver–child bonds in fostering the emotional regulation and exploratory confidence essential for learning [3]. Expanding this view, Bronfenbrenner’s Bioecological Systems Theory conceptualizes development as a product of continuous interactions within a nested set of environmental systems, from the immediate family microsystem to broader socioeconomic and cultural macrosystems [4]. Together, these frameworks mandate a holistic approach, suggesting that the most effective interventions must not only address specific developmental skills but also support the relational and environmental contexts in which a child grows. Consequently, early intervention services for children aged 0–3 have become a cornerstone of global public health and educational policy, aiming to optimize child and family outcomes through structured supports delivered across home, clinical, and community settings.

Synthesizing these perspectives provides a multilevel framework of early development in which children’s developmental outcomes are shaped by interactions among biological dispositions, relational experiences, and environmental contexts. From this perspective, the effects of early intervention depend not only on whether an intervention is provided, but also on how developmental experiences are structured and delivered. Specifically, intervention setting shapes the immediate microsystem in which intervention experiences occur, provider characteristics influence how relational and developmental experiences are delivered, and intervention duration affects the extent and consistency of children’s exposure to intervention, which may be important for experience-dependent developmental change. Population risk status is also relevant because children with different developmental vulnerabilities may respond differently to the same intervention. Furthermore, these perspectives support examining development across multiple domains rather than treating individual developmental outcomes in isolation. Physical growth reflects an important dimension of early development that may be closely intertwined with children’s capacity to engage with and benefit from developmental experiences, while motor, cognitive, and language development represent complementary aspects of functional neurodevelopment. Evaluating physical growth alongside motor, cognitive, and language outcomes therefore provides a conceptual rationale for examining both somatic and neurodevelopmental trajectories following early intervention. Taken together, these theoretical considerations support examining intervention setting, provider characteristics, intervention duration, and population risk status as potential moderators, and evaluating intervention effects across physical growth and motor, cognitive, and language development outcomes.

### 1.1. Gaps in the Current Evidence Synthesis

Despite a broad consensus on their importance, the empirical evidence for early intervention is marked by significant variability and unresolved questions. While numerous systematic reviews have attempted to synthesize this evidence, they suffer from four critical limitations that this meta-analysis is designed to address: (1) a fragmented, population-specific focus; (2) an uncritical pooling of widely varying intervention durations; (3) a failure to systematically identify moderators of effectiveness; and (4) a disconnect between neurodevelopmental and physical outcomes.

First, the existing synthesis literature is highly compartmentalized. A substantial body of research, exemplified by the updated Cochrane review, has focused almost exclusively on post-discharge interventions for preterm or very-low-birth-weight (VLBW) infants [5]. While invaluable, this work reveals that while early programs may improve short-term cognitive and motor outcomes, these gains often fade by preschool age, suggesting a need to understand the factors driving sustainability. Other syntheses target different, narrowly defined clinical cohorts. For example, the “Project AIM” meta-analysis on interventions for young autistic children found that once methodological biases were controlled for, the effects of many popular interventions became non-significant [6]. By focusing on single-risk populations, these reviews fail to provide a comparative, cross-diagnostic perspective. This leaves a critical gap in our understanding of whether certain intervention principles are universally effective or whether they must be tailored to specific risk profiles, such as prematurity, established neurodevelopmental disorders, or socioeconomic disadvantage.

Second, existing reviews have been unable to adequately explain the high heterogeneity observed in intervention outcomes. The question of “what works, for whom, and under what conditions” remains largely unanswered. An overview of systematic reviews on interventions for infants at risk of cerebral palsy, for example, noted that over half of the included trials failed to show superiority over standard care, leaving the optimal dosage, delivery setting (home vs. clinic), and program duration unresolved [7]. This uncertainty is a major barrier to evidence-based practice, as clinicians and policymakers lack clear guidance on how to design and commission the most effective and resource-efficient programs. Without a systematic analysis of these moderating factors, the field is left with a confusing landscape of conflicting results.

Finally, the literature consistently overlooks the crucial interplay between physical and neurodevelopmental outcomes. Most syntheses artificially isolate cognitive, motor, and language domains from the somatic and physiological foundations that support them. Even when physical growth is considered, as in a recent network meta-analysis on premature infants [8], the evidence is described as fragmented and unsystematic. This is a significant oversight, as a child’s physical well-being, including growth, nutrition, and physiological regulation, is inextricably linked to their capacity for higher-order learning and behavioral adaptation. A holistic understanding of development requires an integrated analysis of both body and mind.

### 1.2. The Current Study: A Transdiagnostic and Moderated Approach

The preceding review highlights that the effectiveness of early intervention is not a settled question but a complex puzzle, characterized by high variability and a lack of clear guidance. The field’s central research problem is no longer whether to intervene, but how to move beyond a “one-size-fits-all” model. To address this, the current meta-analysis was designed to overcome the limitations of prior work by systematically untangling the heterogeneity in outcomes.

Our investigation is guided by the principle that effective intervention design requires understanding how and for whom programs work best. We operationalize this through three core strategies. First, we employ a transdiagnostic framework, moving beyond the siloed, population-specific focus of previous reviews. By including a wide range of high-risk infant populations within a single analytical model, we can explore potentially shared implementation characteristics across heterogeneous high-risk populations that transcend specific diagnostic categories.

Second, we aim to explain why outcomes vary by conducting a robust moderator analysis. We hypothesize that the efficacy of an intervention is not inherent but is shaped by its design and implementation. Therefore, we will perform rigorous subgroup analyses to isolate the “active ingredients” of successful programs, focusing on key variables such as intervention duration, delivery setting (home vs. clinic), and provider.

Finally, we recognize that development is an integrated process. We will provide a holistic synthesis by examining outcomes across both neurodevelopmental (motor, cognitive, language) and physical (growth) domains. This approach resists the artificial separation of mind and body common in previous reviews and offers a more comprehensive assessment of intervention impact. Through this multi-faceted approach, we aim to transform a landscape of confusing findings into a clear, evidence-based guide for the field.

## 2. Methodology

### 2.1. Design

This systematic review and meta-analysis was conducted in accordance with JBI methodology for systematic reviews of effectiveness [9] and reported based on the Preferred Reporting Items for Systematic Reviews and Meta-Analyses (PRISMA) 2020 Statement [10] (Supplementary Document S1. PRISMA Checklist). The study protocol was registered in the PROSPERO database (registration number: CRD420261436569). A comprehensive search and review of the literature was conducted across PubMed, Web of Science, Scopus, CINAHL, and PsycINFO. The complete search strategies can be found in Supplementary Document S2.

### 2.2. Study Selection

Following the search, all identified articles were collated in EndNote [11] and uploaded into online systematic review software, Covidence [12]. Two authors independently screened titles and abstracts against the inclusion criteria, followed by the full text review. Inter-reviewer agreement statistics (e.g., Cohen’s κ) were not calculated; disagreements were resolved through discussion and consensus and, if necessary, reviewed by the other author to reach a final decision. Studies were included if they: (1) adopted randomized controlled trials (RCTs), cluster RCTs, or quasi-RCTs as the study design; (2) focused on children aged 0–3 years at the initiation of the intervention; (3) included a control group (standard care, waitlist control, no intervention, or usual community care); (4) reported extractable quantitative data on child developmental outcomes; (5) were written in English; and (6) were published as peer-reviewed journal articles. Studies were excluded if they: (1) adopted non-randomized or observational study designs that were not RCTs, cluster RCTs, or quasi-RCTs; (2) included children older than 3 years at the initiation of the intervention or irrelevant populations; (3) lacked an independent control group for comparison; (4) failed to report extractable quantitative data on child developmental outcomes; (5) were written in a language other than English; or (6) were published as grey literature, abstracts, conference proceedings, dissertations, preprints, or other non-peer-reviewed reports. Furthermore, if papers were published using the same sample, only the main paper on the original study data was included for further meta-analysis. Figure 1 details the results of the search, and the inclusion process was reported in full according to the Preferred Reporting Items for Systematic Reviews and Meta-analyses (PRISMA) flow diagram [10].

### 2.3. Data Extraction

After selection, the included articles were extracted using a data extraction form, including general information: author(s), publication year, region(s), study design, and aim(s); participant characteristics: age, gender, sample size, and developmental risk status; IG: setting, service provider(s), duration/frequency, and curriculum/activities; CG; and findings and limitations/barrier. For studies with multiple follow-up time points, data from the immediate post-intervention assessment were prioritized for primary analysis, with follow-up data included in subgroup analyses if available. Data were extracted independently by two reviewers, with any disagreements resolved by a third reviewer.

### 2.4. Risk of Bias Assessment

The Cochrane risk-of-bias tool for randomized controlled trials (RoB 2.0) [13] was used to assess the risk of bias in included RCTs. Rather than using Intention-to-Treat (ITT) and Per-Protocol (PP) as alternative versions of the tool, each trial was assessed based on its specific targeted estimand: either the effect of assignment to intervention (ITT effect) or the effect of adhering to intervention (per-protocol effect), corresponding strictly to the relevant RoB 2 signalling questions. The domains assessed include randomization process, deviations from intended interventions, missing outcome data, measurement of the outcome, and selection of the reported result. Each study was rated as “high risk”, “low risk” or “some concerns”. Discrepancies in risk of bias ratings were resolved through consensus discussion or third-party adjudication. A summary risk of bias table and figure were generated to present the overall quality of included studies. The results were visualized as a “traffic light” plot using the Risk-of-bias VISualization (robvis) [14]. The single quasi-RCT [15] was evaluated using the RoB 2 framework, with its allocation procedure categorized as raising ‘some concerns’.

### 2.5. Data Synthesis and Statistical Analysis

Statistical analyses were performed using Review Manager (RevMan) software (Version 7.2) [16] and Stata (Version 15.0) [17]. The results measured immediately after intervention were used for data synthesis. For studies that did not directly report the mean and standard deviation (SD), validated statistical transformation methods were employed to ensure data consistency. For studies reporting the median with range and sample size, the mean and SD were estimated using established formulas [18]. For studies reporting the mean with 95% confidence intervals (CIs) and sample size, the SD was calculated according to the methodology provided in the Cochrane Handbook for Systematic Reviews of Interventions [19]. For the one study with multiple intervention groups [20], we selected the combined intervention arm (nutrition education plus multi-nutrient biscuits (MNBs)) for the primary analysis, as it represented the most comprehensive intervention model. We acknowledge that this post hoc selection may introduce a positive bias; however, a sensitivity analysis using the nutrition education arm alone yielded a smaller but directionally consistent effect, suggesting our main finding is robust. For continuous developmental outcomes, Hedges’ g was calculated as the standardized mean difference (SMD) between intervention and control groups, with 95% confidence intervals (CIs) to quantify the magnitude of intervention effects. Hedges’ g values of 0.2, 0.5, and 0.8 were interpreted as small, moderate, and large effects, respectively. Given that combining diverse risk cohorts and interventions assumes early neurodevelopmental plasticity responds to common features of environmental enrichment across diagnoses, overall pooled SMDs serve primarily as descriptive benchmarks, with clinical inferences driven by domain-specific and subgroup analyses. A random-effects model was used for all meta-analyses to account for expected heterogeneity across studies due to variations in intervention design, participant characteristics, and outcome measures. Heterogeneity was assessed using the I^2^ statistic, with I^2^ values of 0% to 40% indicating the heterogeneity might not be important, and values of 30% to 60%, 50% to 90%, and 70% to 100% indicating the included studies may represent moderate, substantial and considerable heterogeneity, respectively [21]. Subgroup analyses were conducted to explore potential sources of heterogeneity across clinical and methodological variables, including intervention setting, provider expertise, population risk status, and intervention duration. To guard against spurious subgroup findings and type I error inflation, formal tests for subgroup interactions were systematically performed across all outcomes. Furthermore, all non-randomized subgroup comparisons were explicitly classified as exploratory rather than confirmatory. For the included cluster RCT [20], sensitivity analysis yielded a directionally consistent estimate (SMD = 0.49, 95% CI: −0.38, 1.36 vs. primary SMD = 0.55, 95% CI: 0.09, 1.01), confirming overall stability despite attenuated significance (*p* = 0.27, *n* = 2) due to reduced power.

### 2.6. Bias and Certainty Assessment Framework

For outcome measures with at least 10 studies included, publication bias was assessed using funnel plots and Egger’s linear regression test. Statistical significance was set at *p* < 0.05. Meanwhile, all developmental outcomes were assessed for evidence certainty via the GRADEpro GDT tool [22]. Starting from high baseline certainty for randomised trials, five domains were evaluated sequentially: risk of bias, inconsistency, indirectness, imprecision, and other considerations. Each outcome was downgraded for identified limitations and rated as high, moderate, low, or very low certainty. A Summary of Findings table was generated to tabulate pooled effects and final GRADE ratings.

## 3. Results

### 3.1. General Information of Included Studies

As detailed in Table 1, a total of 17 studies published between 2010 and 2025 were included in this review. All studies adopted experimental designs, including 15 randomized controlled trials (RCTs) [23,24,25,26,27,28,29,30,31,32,33,34,35,36,37], 1 cluster RCT [20], and 1 quasi-RCT [15]. Geographically, studies were conducted across 10 countries (Figure 2), with the largest numbers originating from Turkey (*n* = 3) [15,23,24] and the United States (*n* = 3) [26,32,33], followed by India (*n* = 2) [34,37], the Netherlands (*n* = 2) [25,29], and the Republic of Korea (*n* = 2) [27,36], and single studies from China [28], France [30], Mongolia [31], Israel [35], and Indonesia [20]. The primary aims of these trials focused on evaluating the efficacy or applicability of targeted early developmental, psychomotor, cognitive, nutritional, or behavioral interventions on multi-domain functional trajectories in high-risk or standard-risk children.

### 3.2. Population Characteristics of Included Studies

The baseline characteristics of the study populations are presented in Table 2. Study populations covered infants, toddlers, and young children aged from birth to 36 months. Corrected age was widely used for preterm samples, ranging from 0 to 24 months [23,24,25,33]. Gender distribution was relatively balanced across most cohorts, with the percentage of female participants hovering around 40% to 65% in most studies, though only 16.7% of participants in a trial on autism spectrum disorder (ASD) were female [32], and a study on neurodevelopmental impairment in preterm infants did not report the gender of the participants [28]. Sample sizes varied extensively, ranging from small-scale pilot designs (*n* = 19) [26] to a large-scale community trial (*n* = 1100) [31]. Regarding developmental risk conditions, participants could be grouped into three major population categories. The first group consisted of preterm and low-birth-weight infants, including very preterm or very-low-birth-weight newborns and those presenting abnormal general movements or bronchopulmonary dysplasia [20,23,29,30,34,37]. The second group covered children facing high risks of neurodevelopmental conditions such as cerebral palsy, ASD, and severe cognitive impairment [24,25,26,27,28,30,32,33]. The final category included individuals with special medical or socioeconomic vulnerabilities, including cleft lip and palate, visual impairment, feeding difficulties, undernutrition, and disadvantaged socioeconomic backgrounds [15,29,35,36].

### 3.3. Service Characteristics of Included Studies

The service characteristics of experimental and control groups are comprehensively synthesized in Table 3. Early interventions were implemented in two primary settings: home-based environments (*n* = 7) [15,23,24,25,30,31,33] or professional clinical settings, including NICUs, university clinics, classroom settings, and specialized hydrotherapy pools (*n* = 10) [20,26,27,28,29,32,34,35,36,37].

Service was provided by a highly specialized, multidisciplinary workforce, including physiotherapists [23,24,25,29,35,37], speech–language pathologists [26], occupational therapists [27,36], nurses [28,34], psychomotor therapists [30], neonatologists [34], pharmacists [34], hydrotherapists [35], cadres [20], and research scientists [15,32]. Meanwhile, several home-based plans relied heavily on therapist-coached or supervised parents [24,28,31,33] to act as primary intervention providers. Intervention durations ranged from 4 weeks [33] to 1 year [25], with session frequencies from daily [20,24,28,31,33] to twice weekly [15,23,26,27] or weekly [20,25,30,35]. Seven interventions lasted less than six months [15,27,33,35,36,37], and 10 lasted six or more than six months [20,23,25,26,28,29,30,31,32,34].

Program curricula spanned family-centered coaching [15,23,25,29], sensory and activity-based motor training [24,33,37], naturalistic communication intervention [26,32], tablet-based cognitive training [27], multisensory stimulation [28], traditional swaddling [31], aquatic motor therapy [30,35], sensory feeding treatment [36], and nutritional education [20,34]. In contrast, control conditions mainly consisted of conventional care or a waitlist.

### 3.4. Findings and Limitations/Barriers of Included Studies

As summarized in Table 4, interventions produced heterogeneous effects on motor development across included studies. Family-based coaching [23], SAFE therapy [24], and hospital-delivered early developmental programs [29,37] significantly improved gross or fine motor performance. Psychomotor intervention yielded favorable short-term motor gains at nine months, especially for infants born to less educated mothers, yet these benefits failed to persist until 24 months [30]. Neuroscience-driven sensorimotor training effectively enhanced unilateral upper-extremity motor capacity in children with asymmetric cerebral palsy [33], and aquatic therapy promoted motor competence and water adaption among visually impaired toddlers [35]. Comparable motor improvements were found between COPCA and routine standard physiotherapy [25], while traditional prolonged tight swaddling exerted neither positive nor adverse impacts on infant psychomotor development [31].

Multiple intervention approaches generated varying cognitive benefits in high-risk young children. Home-centered cognitive training [24] and tablet-based cognitive training [27] significantly boosted overall cognitive performance. The Infant Behavioral Assessment and Intervention Program (IBAIP) induced measurable cognitive improvements only in the subgroup of infants with bronchopulmonary dysplasia rather than the whole study population [29]. In the comparative trial of SAFE versus conventional NDT rehabilitation, conventional NDT was superior in facilitating cognitive advancement [24], and social supplementary curriculum for autistic toddlers contributed to persistent developmental gains relevant to cognitive–social functioning [32].

Language-related improvements were mainly derived from targeted communication interventions. The EMT + PE naturalistic speech intervention markedly elevated receptive and expressive language proficiency as well as speech articulation in children with cleft lip and palate [26], whereas no statistically significant between-group difference in language development was identified when contrasting SAFE and conventional NDT protocols [24]. Social-focused supplementary intervention for autism also indirectly facilitated children’s interactive language development [32].

All feeding behavior, nutrition and body development indicators were pooled within physical growth-related findings. Sensory feeding therapy effectively optimized mealtime behaviors among toddlers with food refusal without significant changes in anthropometric indices [36]. Early enteral sodium supplementation accelerated in-hospital weight gain and decreased the need for rescue hyponatraemia treatment in very preterm infants [34], and combined nutrition education plus multi-nutrient biscuit supplementation successfully alleviated underweight status and reduced the incidence of iron deficiency anemia among undernourished young children [20].

Furthermore, these studies identified several recurring methodological limitations and operational barriers that may impact the validity and generalizability of the pooled effects. First, sample and design constraints consisted of small sample sizes, single-center designs, baseline imbalances, and pervasive shortages of valid negative control groups or long-term neurodevelopmental follow-up protocols [15,25,26,27,28,29,30,32,33,34,35,36,37]. Second, field barriers involved cumbersome logistics for home visits, inconsistent home assessment settings, and fluctuating parental compliance with therapist-led home-based activities [24,33]. Third, substantial confounding and bias risks remained evident, including cross-group contamination effects, coinciding external supplementary treatments, elevated dropout rates within socioeconomically disadvantaged families, and insufficient objective evidence from neuroimaging, EEG testing or standardized progress records to quantify underlying neurological shifts [23,30,31].

### 3.5. Risk of Bias of the Included Studies

The methodological quality and inherent risk of bias of the 17 included studies were evaluated using RoB2 tool (revised version 2019), stratified by target estimand: the effect of assignment to intervention and the effect of adhering to intervention (Figure 3). Among the eight trials assessed for the effect of assignment to intervention, the majority demonstrated a favorable quality profile, exhibiting a low risk of bias or raising only “some concerns”. These studies generally maintained robust performance in the domains of missing outcome data, deviations from intended interventions, and the selection of the reported results. However, a high overall risk of bias was identified in one specific trial, driven by critical methodological vulnerabilities in the measurement of the outcome domain [26]. Additionally, several trials raised some concerns that arose primarily from ambiguities or a lack of clarifying details regarding the underlying randomization process [15,29,37].

For the nine studies assessed for the effect of adhering to intervention, none were classified as having an overall high risk of bias, indicating an acceptable baseline level of methodological rigor across this cohort. Several trials exhibited low risk across all five individual domains [26,28,30,36]. The remaining trials raised some concerns, which were symmetrically distributed between uncertainties regarding the randomization process [23,25,27,33] and potential variations or deviations from the intended intervention protocols [30,32,35].

### 3.6. Meta-Analyses Outcomes

Meta-analyses were performed using random-effects models to pool standardized mean differences (SMDs) across four outcome domains: motor development, cognitive development, language development, and physical growth. High between-study heterogeneity was detected in most domains; sensitivity and subgroup analyses (preterm status, intervention setting, intervention duration) were conducted to explore potential sources of heterogeneity. Given the substantial statistical heterogeneity observed across the pooled syntheses (I^2^ > 90%), pooled overall effect sizes should be interpreted with caution. Accordingly, primary analytical focus was placed on subgroup syntheses to isolate conditional effectiveness and identify specific sources of heterogeneity. The detailed figures are presented in Supplementary Document S3 (Figures S1–S4).

Ten studies evaluated the effects of early interventions on motor development, with a total pool of 814 participants in the intervention group and 827 in the control group. The pooled overall effect demonstrated no statistically significant benefit of early intervention on motor development (SMD = 0.49, 95% CI: −0.12 to 1.10; Z = 1.56, *p* = 0.12), with substantial heterogeneity (τ^2^ = 0.88, χ^2^ = 172.52, df = 9, *p* < 0.00001, I^2^ = 95%). Sensitivity analyses indicated that exclusion of some studies reduced I^2^ to 92% with minimal change in the pooled effect size [29,30]. Subgroup analyses revealed significant motor improvements in professionally led interventions (SMD = 1.16, 95% CI: 0.28 to 2.03; *p* = 0.009, I^2^ = 87%) and interventions less than six months (SMD = 0.68, 95% CI: 0.05 to 1.30; *p* = 0.03, I^2^ = 73%). By contrast, home-based interventions (SMD = 0.05, 95% CI: −0.61 to 0.71, *p* = 0.88, I^2^ = 93%) and interventions lasting six months or longer (SMD = 0.34, 95% CI: −0.56 to 1.25, *p* = 0.46, I^2^ = 97%) did not yield significant effects. When stratified by preterm status, the mixed-risk group showed significant benefits (SMD = 0.38, 95% CI: 0.04 to 0.72; *p* = 0.03, I^2^ = 70%), whereas the preterm subgroup did not (SMD = 0.69, 95% CI: −1.88 to 3.26; *p* = 0.60, I^2^ = 99%). Formal tests for subgroup differences yielded no statistically significant interaction for intervention setting (χ^2^ = 3.91, df = 1, *p* = 0.05, I^2^ = 74.4%), population risk status (χ^2^ = 0.05, df = 1, *p* = 0.82, I^2^ = 0%), or intervention duration (χ^2^ = 0.36, df = 1, *p* = 0.55, I^2^ = 0%). Because subgroup interactions did not reach definitive statistical significance, these subgroup variations represent exploratory observations rather than confirmed moderation effects.

Five studies reported cognitive outcomes, with a total pool of 193 participants in the intervention group and 189 in the control group. The overall synthesized effect indicated that early interventions did not significantly improve cognitive outcomes compared with control conditions (pooled SMD = 0.53, 95% CI: −0.53 to 1.60, Z = 0.98, *p* = 0.33). Heterogeneity among these studies was extremely high and significant (τ^2^ = 1.40, χ^2^ = 89.49, df = 4, *p* < 0.00001, I^2^ = 96%). Exclusion of Alberge et al.’s study reduced I^2^ to 78% [30]. Sequential deletion of the remaining studies caused negligible fluctuations (~1%). Subgroup analysis showed significant cognitive benefits in professionally led interventions (SMD = 0.70, 95% CI: 0.20 to 1.19; *p* = 0.006, I^2^ = 63%), but not in home-based programs (SMD = 0.38, 95% CI: −2.64 to 3.40, *p* = 0.81, I^2^ = 98%). When stratified by intervention duration, interventions lasting less than six months showed a non-significant trend toward benefit (SMD = 1.25, 95% CI: −0.08 to 2.58, *p* = 0.07, I^2^ = 87%), whereas interventions lasting six months or longer showed no significant effect (SMD = 0.08, 95% CI: −1.36 to 1.51, *p* = 0.92, I^2^ = 97%). The test for subgroup interaction between duration strata was not statistically significant (χ^2^ = 1.38, df = 1, *p* = 0.24, I^2^ = 27.4%). No significant differences were found between preterm (SMD = −0.04, 95% CI: −2.21 to 2.14, *p* = 0.97, I^2^ = 98%) and mixed-risk subgroups (SMD = 0.92, 95% CI: −0.02 to 1.87, *p* = 0.06, I^2^ = 85%).

Four studies examined language outcomes, with a total pool of 112 participants in the intervention group and 115 in the control group. The pooled estimate demonstrated a non-significant trend favoring the intervention group (SMD = 0.44, 95% CI: −0.24 to 1.12; Z = 1.28, *p* = 0.20), with moderate-to-high heterogeneity (τ^2^ = 0.38, χ^2^ = 15.96, df = 3, *p* = 0.001, I^2^ = 81%). Removing Kaiser et al.’s study increased I^2^ to 87% [26], while individual removals of other publications caused only trivial changes (~1%). Subgroup analysis by duration demonstrated significant gains in interventions lasting less than six months (SMD = 1.52, 95% CI: 0.86 to 2.19; *p* < 0.0001), compared with interventions lasting six months or longer (SMD = 0.06, 95% CI: −0.24 to 0.35; *p* = 0.71, I^2^ = 0%). The test for subgroup differences yielded a statistically significant interaction (χ^2^ = 15.72, *p* < 0.0001, I^2^ = 93.6%). However, because this shorter-duration subgroup comprises limited trial data, this finding should be interpreted cautiously as an exploratory observation. Stratification by risk status showed that the mixed subgroup had a non-significant benefit (SMD = 0.62, 95% CI: −0.31 to 1.56, *p* = 0.19, I^2^ = 81%) and the preterm subgroup had zero effect (SMD = 0.00, 95% CI: −0.37 to 0.37, *p* = 1.00). Stratification by setting did not demonstrate statistically significant subgroups or modifications in treatment directions for language outcomes (*p* > 0.05).

Three studies assessed physical growth, including a total of 97 participants in the intervention group and 93 in the control group. In contrast to the other domains, early developmental or nutritional interventions exerted a statistically significant beneficial effect on physical outcomes (SMD = 0.55, 95% CI: 0.09 to 1.01; Z = 2.33, *p* = 0.02), with mild-to-moderate heterogeneity (τ^2^ = 0.09, χ^2^ = 4.32, df = 2, *p* = 0.12, I^2^ = 54%). Sensitivity analyses showed that exclusion of Amiti et al.’s study or Kim et al.’s study reduced heterogeneity to 23% [34] and 12% [36], respectively. Subgroup analysis revealed significant benefits among preterm infants (SMD = 0.88, 95% CI: 0.47 to 1.28; *p* < 0.0001), but not in the mixed-risk group (SMD = 0.33, 95% CI: −0.18 to 0.83, *p* = 0.20, I^2^ = 23%). All growth interventions were delivered in professional settings, meaning home-based estimates were not estimable. Sensitivity analysis substituting the combined arm of Ahmad et al. (1.51 ± 0.68 kg) with the single-intervention arm (1.40 ± 0.72 kg) produced an attenuated estimate (Z = 1.79, *p* = 0.07 vs. primary Z = 2.33, *p* = 0.02; I^2^ = 62% vs. 54%). Despite lost statistical significance, the overall direction of effect remained consistent [20].

Given the substantial heterogeneity (I^2^ > 90%) in several analyses, we performed leave-one-out sensitivity analyses for all four overall meta-analyses, excluding one study at a time and recalculating pooled SMD, 95% CI, I^2^, and τ^2^ (Table S2, Supplementary Document S3). Motor and physical growth estimates were robust, whereas cognitive and language estimates were sensitive to individual trials. Notably, exclusion of the largest trial (*n* = 136) [29] changed the cognitive SMD from 0.53 to 0.40 (95% CI: −0.91, 1.71), and exclusion of one trial [24] reduced language I^2^ from 81% to 0%.

### 3.7. Bias and Certainty Assessment Findings

Visual asymmetry was observed in the funnel plot for motor outcomes. However, Egger’s regression test indicated no statistically significant evidence of publication bias or small-study effects (intercept = 1.92, 95% CI: −2.96 to 6.81, t = 0.91, *p* = 0.391). This test has low statistical power with 10 studies; thus, publication bias cannot be definitively excluded. Formal bias testing was not performed for cognitive, language, and physical growth syntheses, each containing fewer than 10 trials. The detailed figures can be found in Supplementary Document S3 (Figures S5 and S6).

The GRADE assessment revealed varying certainty of evidence across the critical developmental outcomes. The certainty for physical growth was graded as moderate, downgraded by one level solely due to imprecision (small cumulative sample size). Motor function yielded low certainty, penalized for serious inconsistency (I^2^ = 95%) and imprecision. Both cognitive and language functions were evaluated as very low certainty, driven concurrently by serious risk of bias in primary trials, substantial inconsistency (I^2^ > 80%), and imprecision stemming from restricted sample sizes and wide 95% confidence intervals. The detailed table can also be found in Supplementary Document S3 (Table S1).

## 4. Discussion

This systematic review and meta-analysis reveals a nuanced and critical landscape for early intervention: effectiveness is not a given but is highly conditional on the intervention’s target, design, and setting. Our findings challenge the notion of a single “best practice” and instead point toward a more tailored, evidence-guided paradigm. However, given the significant statistical heterogeneity (I^2^ > 90%), these pooled overall effect sizes should be interpreted with caution as descriptive benchmarks rather than homogeneous treatment effects. Thus, the clinical relevance of findings is primarily in the subgroup analyses that differentiate conditional effectiveness across different operational settings. The three most significant findings, the distinct success of physical interventions, the association between professionally led or clinic-based interventions and larger pooled effects observed in exploratory subgroup analyses, and the paradoxical effectiveness of shorter programs, collectively provide a roadmap for refining both clinical practice and future research.

### 4.1. Somatic and Growth Outcomes

The most consistent finding was the significant effect of interventions targeting physical growth (SMD = 0.55). This aligns with the foundational tenet of bioecological theory: a child’s development is built upon their immediate biological system [4]. For vulnerable infants, particularly those born preterm, addressing physiological needs like nutritional deficits is not merely a parallel goal but a prerequisite for higher-order developmental progress. The success here lies in the clear, causal pathway: a specific biological deficit (e.g., undernutrition) is met with a direct, targeted input (e.g., nutrient supplementation), leading to a predictable and measurable response. These findings may suggest that addressing physiological needs could support subsequent developmental progress, although evidence remains preliminary. However, these findings must be interpreted with caution. Despite the promising effect size (SMD = 0.55), this analysis included only three studies (*n* = 97 intervention vs. *n* = 93 control), representing a small sample base. Further robust, adequately powered trials are required to confirm these somatic benefits prior to the formulation of broad clinical recommendations.

### 4.2. Evaluation of Delivery Setting and Provider Characteristics

In contrast to physical growth outcomes, the effectiveness of neurodevelopmental interventions was heavily moderated by the delivery context. In exploratory subgroup analyses, clinic-based and professionally delivered interventions were associated with larger point estimates for motor (SMD = 1.16, 95% CI: 0.28 to 2.03, *p* = 0.009) and cognitive outcomes (SMD = 0.70, 95% CI: 0.20 to 1.19, *p* = 0.006).

However, these subgroup patterns should not be interpreted as establishing the causal superiority of clinical settings or professional therapists. Delivery setting and provider expertise are inherently collinear with intervention dosage, structured protocol fidelity, and baseline clinical severity. Crucially, formal tests for subgroup interactions across settings did not reach definitive statistical significance for motor development (χ^2^ = 3.91, df = 1, *p* = 0.05, I^2^ = 74.4%) or cognitive function (χ^2^ = 0.04, df = 1, *p* = 0.84, I^2^ = 0%). Therefore, these numerical differences reflect observational associations rather than proven clinical superiority. Clinical expertise should not replace the natural environment, but should be integrated into a hybrid, blended delivery model that combines structured professional treatment with coached, high-fidelity home routines to support both clinical efficacy and caregiver empowerment.

### 4.3. Intervention Duration as a Potential Effect Modifier

In subgroup comparisons, interventions lasting less than six months exhibited significant language gains (SMD = 1.52, 95% CI: 0.86 to 2.19, *p* < 0.0001), whereas cognitive outcomes showed only a non-significant trend toward benefit (SMD = 1.25, 95% CI: −0.08 to 2.58, *p* = 0.07; χ^2^ = 1.38, df = 1, *p* = 0.24). Because intervention duration was not experimentally assigned or randomized across trials, duration is inherently confounded with baseline developmental risk, intervention intensity, and study design heterogeneity. Therefore, these subgroup patterns should not be interpreted as causal evidence that shorter programs are inherently superior or that concentrated bursts are optimal. Instead, intervention duration should be conceptualized as a conditional effect modifier that warrants prospective validation in randomized head-to-head trials.

### 4.4. Implications for Early Childhood Practice

These findings offer practical implications for early childhood education (ECE) professionals working in inclusive settings. First, educators may consider collaborating with pediatric specialists to embed structured, developmentally targeted activities into daily routines, particularly when such practices are feasible and supported by clinical teams. For example, structured physical activity programs, such as a 12-week mini-trampoline program, have been associated with improvements in motor and executive function among preschool children [38], suggesting that developmentally targeted movement activities may be one feasible component of classroom-based intervention. Second, for premature or low-birth-weight infants, educators should work within multidisciplinary teams to ensure that basic physiological stability and physical growth are supported before prioritizing higher-order cognitive and behavioral learning goals. Finally, when engaging in family-centered or home-visiting initiatives, educators should highlight structured caregiver coaching to improve implementation fidelity and maintain therapeutic dosage within natural environments. When interpreting these findings, the GRADE evidence certainty must be considered, which ranges from moderate for physical growth to low for motor outcomes and very low for cognitive and language outcomes. These certainty ratings also apply to subgroup findings; therefore, the observed advantages of professional-led interventions should be interpreted as preliminary trends rather than definitive practice directives. Observed subgroup advantages for professional-led interventions thus represent preliminary trends rather than definitive directives.

### 4.5. Limitations and Directions for Future Research

The conclusions of this meta-analysis must be considered in light of several limitations, both within our review and in the primary studies themselves. First, adopting a transdiagnostic framework allows us to evaluate whether environmental and structural moderators exert broad effects across clinical phenotypes; however, because different conditions involve distinct underlying mechanisms, synthesized SMDs should be viewed as exploratory benchmark indicators rather than uniform effect sizes for specific therapeutic modalities. Second, by applying strict eligibility criteria, specifically requiring randomized designs and extractable quantitative outcomes, only 17 studies were eligible from over 11,000 screened records. While this selective outcome reflects our prioritization of methodological rigor and homogeneity of exposure over sample volume, it also inherently constrained the sample sizes available for domain-specific subgroup analyses. Third, the included trials were often characterized by small sample sizes, single-center designs, and a lack of standardized outcome measures, which contributes to the high statistical heterogeneity observed. Thus, pooled overall effect sizes should not be interpreted as estimates of a singular intervention effect, but as descriptive summaries that highlight the conditional nature of effectiveness, with meaningful inference being derived from subgroup analyses. In addition, prediction intervals were not estimated because of the limited number of studies within several outcome domains and the instability of such estimates under extreme heterogeneity. This marked heterogeneity may also account for the visual asymmetry observed in the funnel plot for motor outcomes, although quantitative assessment via Egger’s test confirmed no significant publication bias; formal publication bias assessment was unfeasible for cognitive, language and growth analyses owing to fewer than 10 studies per domain, leaving reporting bias risks unquantified for these developmental domains. Finally, all included studies evaluated high-risk or clinically vulnerable infant populations; consequently, findings cannot be generalized to typically developing, healthy infant populations.

Furthermore, several specific methodological constraints apply to our subgroup and moderation findings. Key intervention variables, such as delivery setting, therapist expertise, intervention dosage, and treatment duration, were inherently collinear across the included trials. For instance, professional-led interventions occurred mainly in clinical settings, while parent-mediated ones occurred at home, precluding definitive isolated-variable causal inference. Because intervention characteristics were not experimentally assigned or randomized across studies, observed subgroup differences reflect observational associations rather than confirmed treatment effect modifiers. Additionally, substantial heterogeneity remained across several outcomes even after subgroup stratification, likely reflecting variations in baseline clinical severity and protocol implementation fidelity.

More importantly, these methodological weaknesses point toward a clear agenda for the next generation of early intervention research. First, there is an urgent need for large-scale, multi-center RCTs with standardized protocols and long-term follow-up to assess the sustainability of gains. Second, future studies must move beyond behavioral outcomes alone. The integration of objective neurological measures, such as neuroimaging or electroencephalography (EEG), is critical to understanding the underlying mechanisms of change and identifying which interventions successfully alter brain structure and function. Finally, future research should employ factorial or head-to-head designs to disconfound delivery setting, provider expertise, and treatment duration, thereby validating optimal intervention parameters under rigorous prospective conditions.

## 5. Conclusions

Our meta-analysis evidence suggests that early intervention outcomes are highly context-dependent. Given the substantial statistical heterogeneity observed across pooled syntheses, these conclusions are based on subgroup-specific conditional effects rather than overall pooled estimates. While subgroup observations suggest favorable trends for professionally led models, conclusions remain constrained by low to very low evidence certainty across developmental outcomes. High-quality trials are needed before establishing firm clinical recommendations.

Ultimately, this review argues for a paradigm shift from broad-based programs to precisely tailored interventions. Future progress hinges on our ability to conduct large, standardized longitudinal studies that combine behavioral metrics with objective neuroimaging, allowing us to move from what works to understanding why it works and for whom.

## Figures and Tables

**Figure 1:**
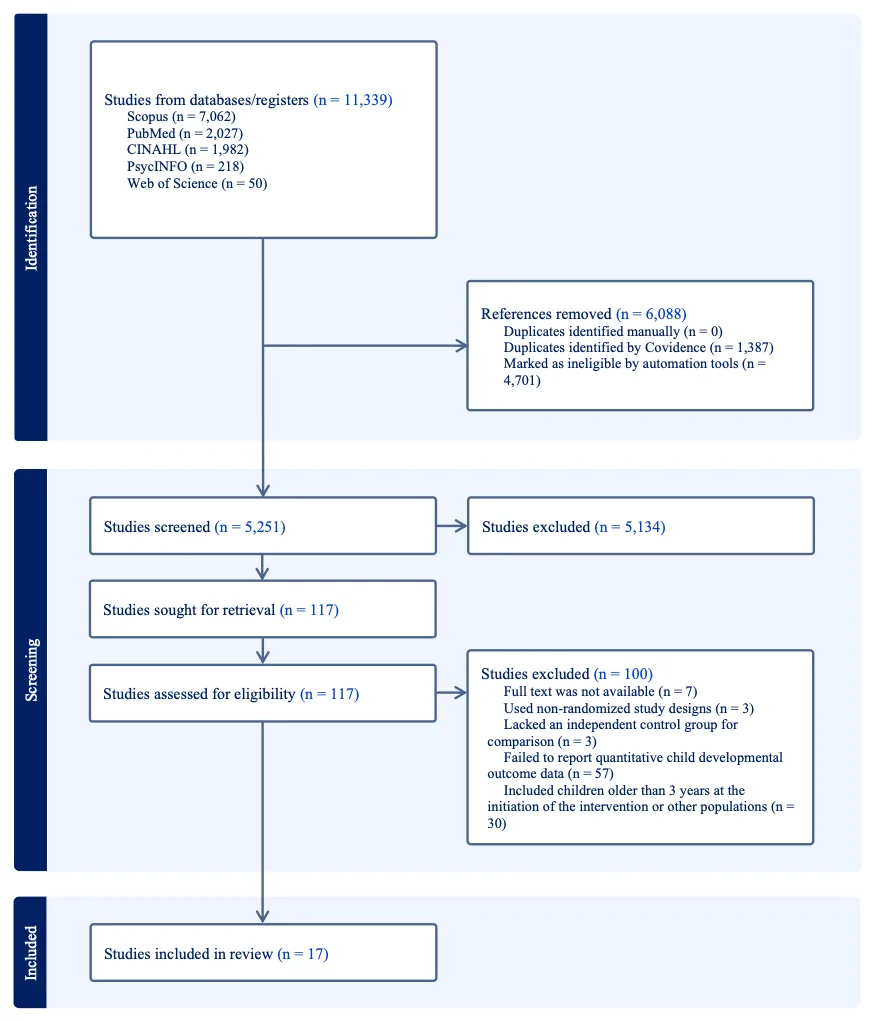
The Selection of Studies Included in This Review.

**Figure 2:**
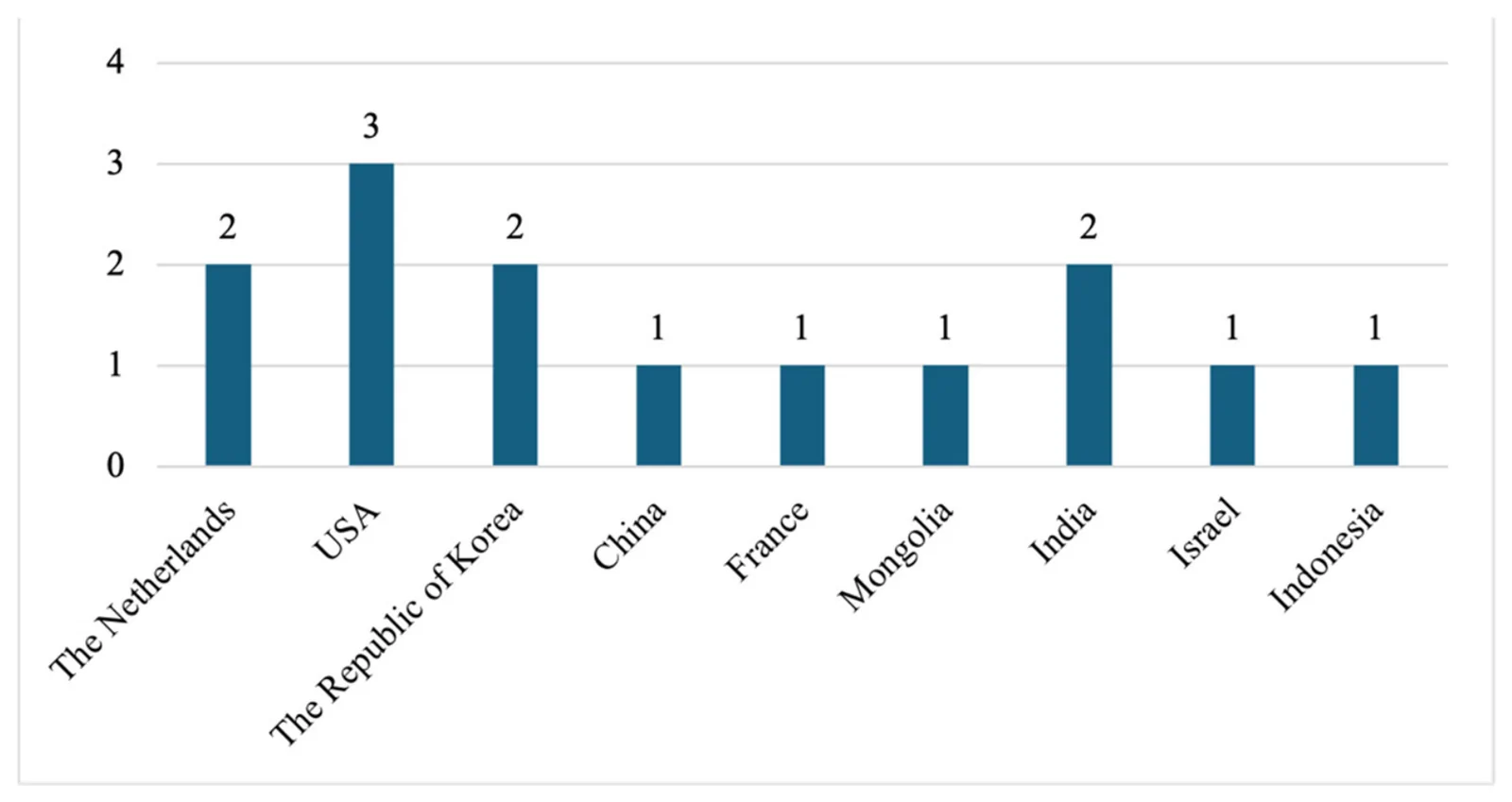
Regions of the Included Studies.

**Figure 3:**
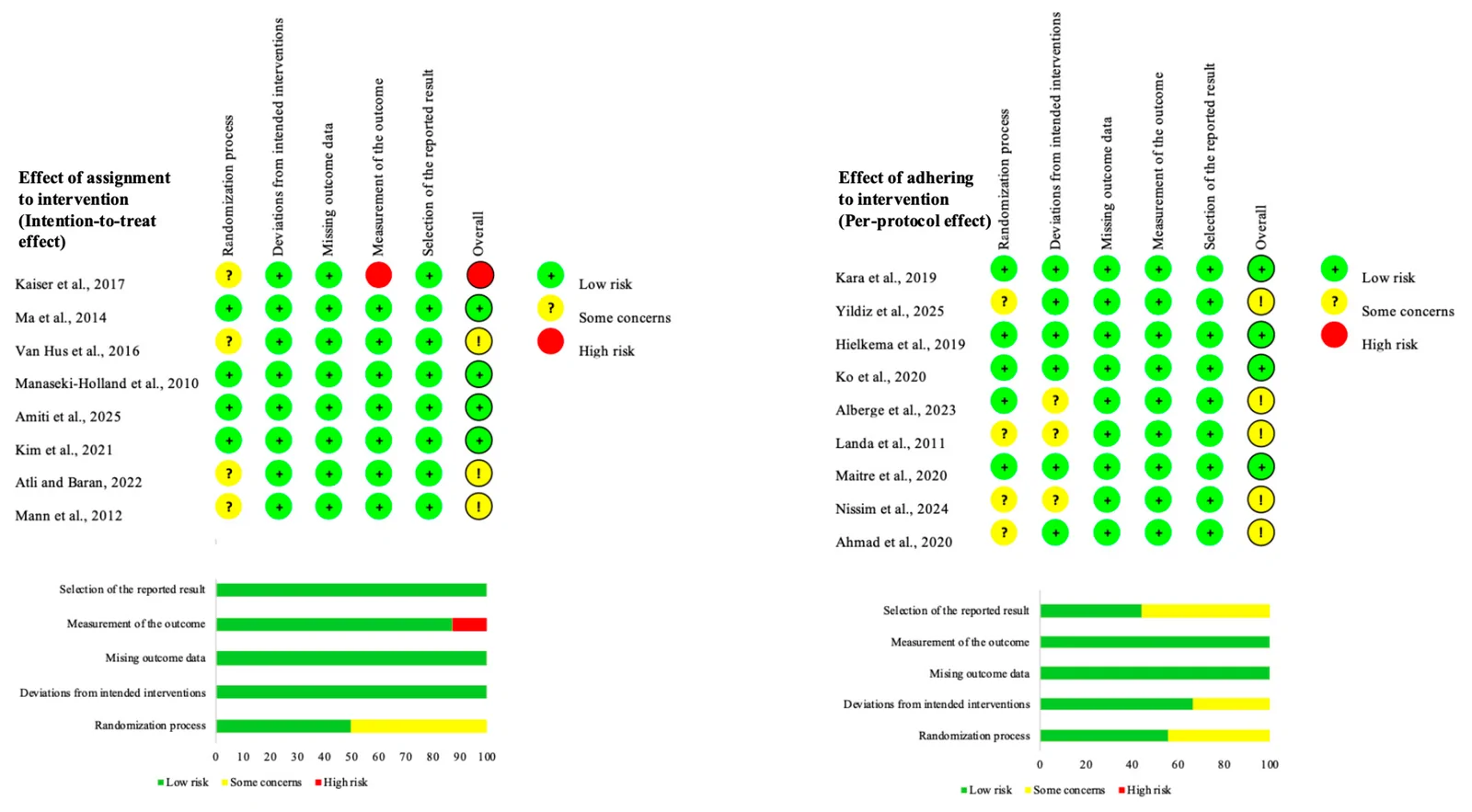
Risk of Bias of the Included Studies [15,20,23,24,25,26,27,28,29,30,31,32,33,34,35,36,37].

**Table 1:** General Information of the Included Studies.

No.	Author(s)/Year	Region(s)	Study Design	Aim
1	Kara et al., 2019 [23]	Turkey	RCT	To examine family-based intervention effects on motor function in preterm infants
2	Yildiz et al., 2025 [24]	Turkey	RCT	To compare SAFE early intervention with conventional NDT for cognitive, language, and motor development in infants at risk of CP
3	Hielkema et al., 2019 [25]	Netherlands	RCT	To compare COPCA intervention with standard physiotherapy in infants at very high risk of CP
4	Kaiser et al., 2017 [26]	United States of America	RCT	To evaluate EMT + PE naturalistic communication intervention for speech–language outcomes in toddlers with CL/P
5	Ko et al., 2020 [27]	Republic of Korea	RCT	To evaluate the applicability and efficacy of tablet computer-based cognitive training for young children with cognitive impairment
6	Ma et al., 2014 [28]	China	RCT	To characterize preterm infants’ GMs and evaluate early intervention effects on GM quality
7	Van Hus et al., 2016 [29]	Netherlands	RCT	To investigate longitudinal effects of the IBAIP on cognitive and motor development of very preterm infants
8	Alberge et al., 2023 [30]	France	RCT	To assess the efficacy of early post-hospital psychomotor therapy on neurodevelopment of very preterm infants
9	Manaseki-Holland et al., 2010 [31]	Mongolia	RCT	To investigate the effects of traditional tight and prolonged swaddling on infant mental and psychomotor development
10	Landa et al., 2011 [32]	United States of America	RCT	To evaluate the impact of supplementing the intervention with a curriculum targeting socially synchronous behavior on social outcomes of toddlers with ASD
11	Maitre et al., 2020 [33]	United States of America	RCT	To test a neuroscience-based, multi-component intervention for somatosensory processing and motor function of the more-affected UE in infants with CP and asymmetric UE impairments
12	Amiti et al., 2025 [34]	India	RCT	To evaluate early enteral sodium supplementation on weight gain velocity in very preterm infants
13	Nissim et al., 2024 [35]	Israel	RCT	To assess combined physiotherapy and aquatic motor intervention for motor skills and water adjustment in visually impaired toddlers
14	Kim et al., 2021 [36]	Republic of Korea	RCT	To compare sensory-based feeding treatment with nutrition education for toddlers with food refusal
15	Ahmad et al., 2020 [20]	Indonesia	Cluster RCT	To investigate nutrition education plus multi-nutrient biscuits for growth and iron deficiency anemia in underweight children
16	Atli and Baran, 2022 [15]	Turkey	Quasi-RCT	To evaluate a home-centered cognitive support program for socioeconomically disadvantaged infants
17	Mann et al., 2012 [37]	India	RCT	To compare hospital-based early developmental intervention with home intervention for motor performance in preterm infants

**Table 2:** Population Characteristics of the Included Studies.

No.	Author(s)/Year	Age	Gender	Sample Size	Developmental Risk Status
1	Kara et al., 2019 [23]	3 months corrected age (CA)	50% female	32 (16; 16)	Preterm, birth weight ≤ 1500 g, abnormal GMs
2	Yildiz et al., 2025 [24]	3 months CA	50% female	26 (23; 23)	At risk of CP, NICU stay ≥ 15 days
3	Hielkema et al., 2019 [25]	0–9 months CA	39.5% female	39 (23; 16)	Very high risk of CP
4	Kaiser et al., 2017 [26]	15–36 months	31.6% female	19 (8; 11)	Nonsyndromic CL/P, typical cognition, at risk of speech–language delays
5	Ko et al., 2020 [27]	18–36 months	52.6% female	38 (20; 18)	Cognitive impairment
6	Ma et al., 2014 [28]	Gestational age (GA): 28 weeks 2 days to 36 weeks 5 days	N.A.	285 (145; 140)	Preterm infants at high risk of neurodevelopmental impairment
7	Van Hus et al., 2016 [29]	<32 weeks GA	48.3% female	136 (69; 67)	Very preterm/VLBW infants, some with BPD and those born to mothers with low education
8	Alberge et al., 2023 [30]	<30 weeks GA	44% female	114 (57; 57)	Very preterm, high neurodevelopmental disability risk
9	Manaseki-Holland et al., 2010 [31]	11–17 months	50.6% female	1100 (536; 564)	N.A.
10	Landa et al., 2011 [32]	21–33 months	16.7% female	48 (24; 24)	ASD
11	Maitre et al., 2020 [33]	6–24 months CA	51% female	73 (37; 36)	CP with asymmetric UE impairment
12	Amiti et al., 2025 [34]	25 weeks–30 weeks + 6 days GA	50.5% female	104 (52; 52)	Very preterm, growth/hyponatremia risk
13	Nissim et al., 2024 [35]	8–36 months	60.6% female	33 (18; 15)	Visual impairment
14	Kim et al., 2021 [36]	12–36 months	65.4% female	26 (14; 12)	At risk of feeding disorders, sensory processing difficulties, and delayed growth
15	Ahmad et al., 2020 [20]	6–23 months	52.1% female	121 (NE: 30; NE + MNB: 31; MNB: 31; Control: 29)	Underweight
16	Atli and Baran, 2022 [15]	18–24 months	56.7% female	30 (15; 15)	Socioeconomically disadvantaged
17	Mann et al., 2012 [37]	<37 weeks GA	37.2% female	30 (15; 15)	Preterm infants at risk of motor delay

**Table 3:** Service Characteristics of the Included Studies.

No.	Author(s)/Year	Setting of IG	Service Provider(s) of IG	Duration of IG	Frequency of IG	Curriculum/Activities of IG	CG
1	Kara et al., 2019 [23]	Home	Pediatric physiotherapist	9 months	Twice weekly	Family-based COPCA	Traditional early intervention
2	Yildiz et al., 2025 [24]	Home	Physiotherapists and parents	12 weeks	Daily	Sensory strategies, activity-based motor training, family collaboration, and environmental enrichment	Conventional NDT home care
3	Hielkema et al., 2019 [25]	Home	COPCA-trained physiotherapists	1 year	Weekly	Family-centered coaching	Typical infant physiotherapy
4	Kaiser et al., 2017 [26]	University clinic	Speech–language pathologists	6 months	Twice weekly	Play-based EMT + PE for speech–language	Business-as-usual (BAU)
5	Ko et al., 2020 [27]	Hospital	Occupational therapists	12 weeks	Twice weekly	Adaptive and non-adaptive cognitive tasks	Traditional rehabilitation
6	Ma et al., 2014 [28]	NICU and clinic	Nurses and parents	3–54 weeks GA	Hospital phase 1–2 times/day, family phase 2–3 times/day + daily hydrotherapy	Multi-sensory stimulation, gymnastics, hydrotherapy	Standard care
7	Van Hus et al., 2016 [29]	Hospital and home	IBAIP-trained physiotherapists	Pre-discharge–6 months CA	1 pre-discharge and 6–8 home sessions	Neurobehavioral support, parent–infant responsive interaction, infant self-regulation facilitation	Standard care
8	Alberge et al., 2023 [30]	Home	Psychomotor therapists	2–9 months CA	Weekly ×4 months, then biweekly ×4 months	Psychomotor therapy	Standard care
9	Manaseki-Holland et al., 2010 [31]	Home	Parents (supervised)	Birth–7 months	Daily	Wrapping infant from head/neck down in cotton cloths and blankets	Usual community care
10	Landa et al., 2011 [32]	Classroom	Trained interventionists	6 months	4 days/week, 2.5 h/day	Supplemental social curriculum layered onto the AEPS curriculum	AEPS only
11	Maitre et al., 2020 [33]	Home	Parents (therapist-coached)	4 weeks	Daily	Bimanual play, UE constraint, sensorimotor training	Waitlist (bimanual play only)
12	Amiti et al., 2025 [34]	Neonatal unit	Neonatologists, nurses, and pharmacists	Birth–34 weeks PMA	8–12×/day with feeds	Enteral sodium 4 mEq/kg/day	Usual care with placebo
13	Nissim et al., 2024 [35]	Hydrotherapy pool	Hydrotherapist and physiotherapists	12 weeks	Weekly	Halliwick aquatic motor intervention and routine physiotherapy	Routine physiotherapy
14	Kim et al., 2021 [36]	Clinic	Occupational therapist (SOS-trained)	12 weeks	5 days/week ×4 weeks, then 1×/week ×8 weeks	SOS sensory feeding intervention	General nutrition education
15	Ahmad et al., 2020 [20]	Community	Trained cadres	6 months	NE weekly, MNB daily	Cadre training, weekly home visits, FMC-based complementary feeding monitoring, booklet/poster education, responsive feeding guidance; daily fortified MNB	Regular nutrition education
16	Atli and Baran, 2022 [15]	Home	Researcher	12 weeks	Twice weekly	Home-based cognitive development activities	Usual community care
17	Mann et al., 2012 [37]	Hospital	Physiotherapist	4 months	Thrice weekly	Prone/side lying positioning, eye following & neck muscle strengthening, supported sitting; shoulder mobilization, and forearm weight bearing for head control	Home intervention

**Table 4:** Findings and Limitations/Barriers of the Included Studies.

No.	Author(s)/Year	Findings	Limitations or Barriers
1	Kara et al., 2019 [23]	Both family-based and traditional early interventions significantly improved fine and gross motor development in preterm infants in the first 2 years; no significant between-group differences.	No neuroimaging/EEG data on brain development; no assessment of mother–infant interaction.
2	Yildiz et al., 2025 [24]	SAFE significantly improved motor and language composite scores by 6 months; motor gains persisted at 12 months; time × group interaction favored SAFE for motor development and NDT for cognitive development; no significant interaction for language.	Rural home-visit logistics; variable home assessment environments.
3	Hielkema et al., 2019 [25]	COPCA and typical infant physiotherapy yielded similar neuromotor, cognitive, and behavioral outcomes; intervention components were not associated with developmental outcomes.	Small sample size; selective attrition in CG; possible contaminating.
4	Kaiser et al., 2017 [26]	EMT + PE improved receptive/expressive language and speech clarity.	Small sample size and baseline imbalance; unstandardized speech measures; constrained assessments; confounding concurrent treatments; limited generalizability.
5	Ko et al., 2020 [27]	Tablet-based cognitive training feasible and effective for cognitive age 18–36 months, improving cognitive, social, attentional, and goal attainment outcomes independent of delay severity; self-care improvement reduced with severe cognitive delay.	Single center; no real-life function assessment, attention-focused measures only; no caregiver satisfaction; no objective measurements to evaluate interest; no performance progress records; no true negative control; no long-term follow-up.
6	Ma et al., 2014 [28]	Lower gestational age/birth weight correlated with higher cramped-synchronized GM rate; early intervention significantly improved fidgety movements but not writhing movements	Short intervention window; high infant sleep requirements limiting intervention time; no AF movement statistical power; no long-term neurodevelopmental follow-up; unclear generalizability
7	Van Hus et al., 2016 [29]	Significant longitudinal improvements in motor development for the intervention group; non-significant longitudinal effect on overall cognitive development; large beneficial effects in both cognitive and motor domains for the BPD subgroup; stable intervention effects maintained over five years.	Missing data from participants not attending all follow-up time points; variable assessment tools; unmeasured selective cognitive gains.
8	Alberge et al., 2023 [30]	No significant difference in BSID-III scores at 24 months; significant improvement in gross motricity at 9 months; significant benefit in fine motricity for infants of educationally underserved mothers at 9 months; no between-group difference in neuromotor function.	Small sample size; heterogeneity of professional skills; contamination effect; non-validated scale; high attrition among socially disadvantaged families.
9	Manaseki-Holland et al., 2010 [31]	Traditional tight prolonged swaddling has no significant adverse or beneficial effects on infant mental and psychomotor development at 13 months; Mongolian infants’ BSID-II scores are comparable to US norms.	Inability to identify characteristics of families who refused recruitment; limited generalizability to rural populations; no high-risk newborns.
10	Landa et al., 2011 [32]	Significant treatment effect for SEI, which was generalized and was maintained; significant time effects for all outcomes across both groups; IS group showed significantly more rapid growth in SEI compared to Non-IS group during intervention.	No monitor-only control group.
11	Maitre et al., 2020 [33]	Intervention improved UE motor function and somatosensory processing; safety demonstrated by lack of negative impact on the constrained limb or gross motor function.	Inconsistent parent adherence; incomplete characterization of somatosensory processing via P200 amplitude; no long-term data; unassessed contributions of individual intervention components; and insufficient statistical power for constraint–dosage analysis.
12	Amiti et al., 2025 [34]	Early enteral sodium supplementation significantly improves in-hospital growth without adverse events; sodium supplementation reduces the requirement for rescue hyponatremia correction.	Unaccounted sodium from milk/fortifiers/drugs; no urine osmolality/urine sodium tests; no external validation of solution sodium content; short follow-up.
13	Nissim et al., 2024 [35]	Aquatic–physiotherapy significantly improved motor skills and water adjustment.	Small sample size; no subgroup analyses by visual impairment type; limited statistical power.
14	Kim et al., 2021 [36]	Sensory feeding significantly improved child and parent mealtime behavior (BPFAS) in food-refusal toddlers; no anthropometric changes.	Small sample size; short duration; no socioeconomic/parent–child interaction data; no subgroup analysis; unclear long-term effects.
15	Ahmad et al., 2020 [20]	NE + MNB significantly reduced underweight and iron deficiency.	N.A.
16	Atli and Baran, 2022 [15]	Home cognitive program significantly improved cognition; effects retained short-term.	Small sample size; short follow-up; limited generalizability; no long-term effect evaluation.
17	Mann et al., 2012 [37]	EIP improved gross motor performance more than home intervention.	Small sample size; no subgrouping of preterm severity; infant non-cooperation; limited resources; no sophisticated detection measures.

## Data Availability

No new data were created or analyzed in this study. Data sharing is not applicable to this article.

## Supplementary Materials

The following supporting information can be downloaded at: https://www.mdpi.com/article/10.3390/children13101324/s1, Supplementary document S1: PRISMA Checklist; Supplementary document S2: Search Strategy. Supplementary document S3: Figure S1. The effects of early interventions on motor development; Figure S2. The effects of early interventions on cognitive development; Figure S3. The effects of early interventions on language development; Figure S4. The effects of early interventions on physical growth; Figure S5. The funnel plot for motor outcomes; Figure S6. The Egger’s regression plot; Table S1. A summary of findings from the certainty assessment; Table S2. Leave-one-out sensitivity analysis for overall meta-analyses.
