# Supplementary material for: The Effectiveness of Early Intervention Programs for Children Aged 0–3: A Systematic Review and Meta-Analysis
Source: Children (Basel). 2026 Sep 29;13(10):1324. doi: 10.3390/children13101324 (PMC13702888; doi:10.3390/children13101324)
Supplement: Supplementary file 1 [file children-13-01324-s001.zip › Supplementary document S2. Search Strategy.pdf]

Supplementary document 2

1. Search Strategy for PubMed

Date searched: 24/02/2026
Filters applied: Full text, randomized controlled trial, English, child: birth-18 years
Results: 2,027

("Infant"[Mesh] OR "Infant, Newborn"[Mesh]) OR ("0-3 years"[tiab] OR "0-36 months"[tiab] OR "birth to three"[tiab] OR "under three"[tiab] OR infant*[tiab] OR newborn*[tiab] OR neonatal[tiab] OR babies[tiab] OR toddler*[tiab] OR "early years"[tiab]))
AND
("Early Intervention, Educational"[Mesh] OR "Home Care Services"[Mesh] OR "Parenting"[Mesh] OR "Family Support"[Mesh] OR "Parent-Child Relations"[Mesh] OR ("early intervention"[tiab] OR "development* intervention*"[tiab] OR "home visit*"[tiab] OR "home visiting program*"[tiab] OR "parent intervention*"[tiab] OR "parent training"[tiab] OR "parent education"[tiab] OR "family support"[tiab] OR "developmental support"[tiab] OR "early stimulation"[tiab] OR "infant stimulation"[tiab] OR "psychosocial support"[tiab]))

2. Search Strategy for Web of Science

Date searched: 24/02/2026
Filters applied: Article, English, all open access.
Results: 3,083

("0-3 years" OR "0-36 months" OR "birth to three" OR "under three" OR "infant" OR "newborn" OR "neonatal" OR "babies" OR "toddler" OR "early years") (Abstract)
AND
("early intervention" OR "development intervention*" OR "home visiting program" OR "parent intervention*" OR "family support" OR "developmental support" OR "early stimulation*") (Abstract) and ("effect*" OR "outcome*" OR "benefit*" OR "impact" OR "efficacy" OR "evaluation" OR "improvement") (Abstract)

3. Search Strategy for Scopus

Date searched: 24/02/2026
Filters applied: Article, English, journal, final, all open access.
Results: 7,062

TITLE-ABS-KEY ("0-3 years" OR "0-36 months" OR "birth to three" OR "under three" OR infant* OR newborn* OR neonatal OR babies OR toddler* OR "early years")
AND
TITLE-ABS-KEY ("early intervention" OR "development* intervention*" OR "home visit*" OR "home visiting program*" OR "parent intervention*" OR "parent training"

OR "parent education" OR "family support" OR "developmental support" OR "early stimulation" OR "infant stimulation" OR "psychosocial support")

4. Search Strategy for CINAHL (via EBSCOhost)
Date searched: 24/02/2026
Filters applied: English, infant, newborn: birth-1 month, child, preschool: 2-5 years, infant: 1-23 months, all infant, all child, full text, peer-reviewed.
Results: 1,982

((MH "Infant+") OR (MH "Newborn+") OR (TI ("0-3 years" OR "0-36 months" OR "birth to three" OR "under three" OR infant* OR newborn* OR neonatal OR babies OR toddler* OR "early years") OR AB ("0-3 years" OR "0-36 months" OR "birth to three" OR "under three" OR infant* OR newborn* OR neonatal OR babies OR toddler* OR "early years"*)))
AND
((MH "Early Intervention+") OR (MH "Home Visiting+") OR (MH "Parent Education+") OR (MH "Family Support+") OR (TI ("early intervention" OR "development* intervention*" OR "home visit*" OR "parent intervention*" OR "parent training" OR "parent education" OR "family support" OR "developmental support" OR "early stimulation" OR "infant stimulation" OR "psychosocial support") OR AB ("early intervention" OR "development* intervention*" OR "home visit*" OR "parent intervention*" OR "parent training" OR "parent education" OR "family support" OR "developmental support" OR "early stimulation" OR "infant stimulation" OR "psychosocial support")))

5. Search Strategy for PsycINFO (via EBSCOhost)
Date searched: 24/02/2026
Filters applied: Journal, English, neonatal (birth-1 mo), preschool age (2-5 yrs), infancy (2-23 mo), childhood (birth-12 yrs), empirical study, quantitative study, interview, longitudinal study, qualitative study, followup study, clinical trial, prospective study, focus group, treatment outcome, retrospective study, clinical case study, field study, peer-reviewed, open access.
Results: 218

((DE "Infants") OR (DE "Neonates") OR (TI ("0-3 years" OR "0-36 months" OR "birth to three" OR "under three" OR infant* OR newborn* OR neonatal OR babies OR toddler* OR "early years") OR AB ("0-3 years" OR "0-36 months" OR "birth to three" OR "under three" OR infant* OR newborn* OR neonatal OR babies OR toddler* OR "early years")))
AND
((DE "Early Intervention") OR (DE "Parent Training") OR (DE "Parent Education") OR (DE "Family Programs") OR (TI ("early intervention" OR "development* intervention*" OR "home visit*" OR "parent intervention*" OR "parent training" OR "parent education" OR "family support" OR "developmental support" OR "early

stimulation" OR "infant stimulation" OR "psychosocial support") OR AB ("early intervention" OR "development* intervention*" OR "home visit*" OR "parent intervention*" OR "parent training" OR "parent education" OR "family support" OR "developmental support" OR "early stimulation" OR "infant stimulation" OR "psychosocial support")))
