# Supplementary material for: The Effectiveness of Early Intervention Programs for Children Aged 0–3: A Systematic Review and Meta-Analysis
Source: Children (Basel). 2026 Sep 29;13(10):1324. doi: 10.3390/children13101324 (PMC13702888; doi:10.3390/children13101324)
Supplement: Supplementary file 1 [file children-13-01324-s001.zip › Supplementary document S3. Figures & tables.pdf]

Figure S1. The effects of early interventions on motor development

Study or Subgroup	Intervention Mean	Intervention SD	Intervention Total	Control Mean	Control SD	Control Total	Weight	Std. Mean Difference IV, Random, 95% CI
Alberge et al., 2023	91.4	1.3	57	93.6	1.4	57	10.5%	−1.62 [−2.04, −1.19]
Hielkema et al., 2019	81.75	5.82	23	80	4.66	16	9.8%	0.32 [−0.32, 0.96]
Kara et al., 2019	4	2.06	16	3.87	2.65	16	9.6%	0.05 [−0.64, 0.75]
Ko et al., 2020	50.7	4.9	20	45.3	9.3	18	9.8%	0.72 [0.06, 1.38]
Maitre et al., 2020	11.7	5.7	37	10.1	6.3	36	10.4%	0.26 [−0.20, 0.72]
Manaseki-Holland et al., 2010	100.34	14.31	536	99.64	15.79	564	11.0%	0.05 [−0.07, 0.16]
Mann et al., 2012	14.33	1.02	15	12.43	1.02	15	9.0%	1.81 [0.94, 2.68]
Nissim et al., 2024	156.66	40	18	149.4	45.12	15	9.7%	0.17 [−0.52, 0.85]
Van Hus et al., 2016	0.12	0.12	69	−0.12	0.13	67	10.5%	1.91 [1.50, 2.32]
Yildiz et al., 2025	107.82	11.78	23	89.52	14.05	23	9.8%	1.39 [0.74, 2.04]
**Total (95% CI)**			**814**			**827**	**100.0%**	**0.49 [−0.12, 1.10]**

Heterogeneity: $Tau^2 = 0.88$; $Chi^2 = 172.52$, df = 9 ($P < 0.00001$); $I^2 = 95\%$
Test for overall effect: Z = 1.56 (P = 0.12)

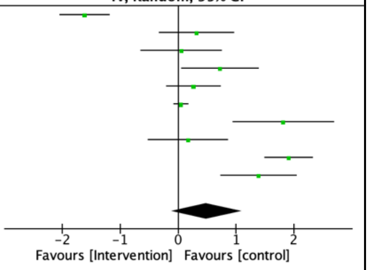

1.1. Subgroup analysis of the mixed group and the preterm group

Study or Subgroup	Intervention Mean	Intervention SD	Intervention Total	Control Mean	Control SD	Control Total	Weight	Std. Mean Difference IV, Random, 95% CI
**1.1.1 Mixed**								
Hielkema et al., 2019	81.75	5.82	23	80	4.66	16	9.8%	0.32 [−0.32, 0.96]
Kara et al., 2019	4	2.06	16	3.87	2.65	16	9.6%	0.05 [−0.64, 0.75]
Ko et al., 2020	50.7	4.9	20	45.3	9.3	18	9.8%	0.72 [0.06, 1.38]
Maitre et al., 2020	11.7	5.7	37	10.1	6.3	36	10.4%	0.26 [−0.20, 0.72]
Manaseki-Holland et al., 2010	100.34	14.31	536	99.64	15.79	564	11.0%	0.05 [−0.07, 0.16]
Nissim et al., 2024	156.66	40	18	149.4	45.12	15	9.7%	0.17 [−0.52, 0.85]
Yildiz et al., 2025	107.82	11.78	23	89.52	14.05	23	9.8%	1.39 [0.74, 2.04]
**Subtotal (95% CI)**			**673**			**688**	**70.0%**	**0.38 [0.04, 0.72]**
Heterogeneity: $Tau^2 = 0.13$; $Chi^2 = 20.11$, df = 6 (P = 0.003); $I^2 = 70\%$								
Test for overall effect: Z = 2.21 (P = 0.03)								
**1.1.2 Preterm**								
Alberge et al., 2023	91.4	1.3	57	93.6	1.4	57	10.5%	−1.62 [−2.04, −1.19]
Mann et al., 2012	14.33	1.02	15	12.43	1.02	15	9.0%	1.81 [0.94, 2.68]
Van Hus et al., 2016	0.12	0.12	69	−0.12	0.13	67	10.5%	1.91 [1.50, 2.32]
**Subtotal (95% CI)**			**141**			**139**	**30.0%**	**0.69 [−1.88, 3.26]**
Heterogeneity: $Tau^2 = 5.08$; $Chi^2 = 149.48$, df = 2 ($P < 0.00001$); $I^2 = 99\%$								
Test for overall effect: Z = 0.53 (P = 0.60)								
**Total (95% CI)**			**814**			**827**	**100.0%**	**0.49 [−0.12, 1.10]**

Heterogeneity: $Tau^2 = 0.88$; $Chi^2 = 172.52$, df = 9 ($P < 0.00001$); $I^2 = 95\%$
Test for overall effect: Z = 1.56 (P = 0.12)
Test for subgroup differences: $Chi^2 = 0.05$, df = 1 (P = 0.82), $I^2 = 0\%$

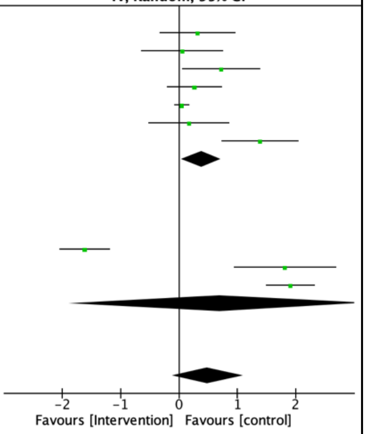

1.2. Subgroup analysis of the professional group and the home group

Study or Subgroup	Intervention Mean	Intervention SD	Intervention Total	Control Mean	Control SD	Control Total	Weight	Std. Mean Difference IV, Random, 95% CI
**2.1.1 Professional**								
Ko et al., 2020	50.7	4.9	20	45.3	9.3	18	9.8%	0.72 [0.06, 1.38]
Mann et al., 2012	14.33	1.02	15	12.43	1.02	15	9.0%	1.81 [0.94, 2.68]
Nissim et al., 2024	156.66	40	18	149.4	45.12	15	9.7%	0.17 [−0.52, 0.85]
Van Hus et al., 2016	0.12	0.12	69	−0.12	0.13	67	10.5%	1.91 [1.50, 2.32]
**Subtotal (95% CI)**			**122**			**115**	**38.9%**	**1.16 [0.28, 2.03]**
Heterogeneity: $Tau^2 = 0.68$; $Chi^2 = 23.20$, df = 3 ($P < 0.0001$); $I^2 = 87\%$								
Test for overall effect: Z = 2.59 (P = 0.009)								
**2.1.2 Home**								
Alberge et al., 2023	91.4	1.3	57	93.6	1.4	57	10.5%	−1.62 [−2.04, −1.19]
Hielkema et al., 2019	81.75	5.82	23	80	4.66	16	9.8%	0.32 [−0.32, 0.96]
Kara et al., 2019	4	2.06	16	3.87	2.65	16	9.6%	0.05 [−0.64, 0.75]
Maitre et al., 2020	11.7	5.7	37	10.1	6.3	36	10.4%	0.26 [−0.20, 0.72]
Manaseki-Holland et al., 2010	100.34	14.31	536	99.64	15.79	564	11.0%	0.05 [−0.07, 0.16]
Yildiz et al., 2025	107.82	11.78	23	89.52	14.05	23	9.8%	1.39 [0.74, 2.04]
**Subtotal (95% CI)**			**692**			**712**	**61.1%**	**0.05 [−0.61, 0.71]**
Heterogeneity: $Tau^2 = 0.61$; $Chi^2 = 76.04$, df = 5 ($P < 0.00001$); $I^2 = 93\%$								
Test for overall effect: Z = 0.15 (P = 0.88)								
**Total (95% CI)**			**814**			**827**	**100.0%**	**0.49 [−0.12, 1.10]**

Heterogeneity: $Tau^2 = 0.88$; $Chi^2 = 172.52$, df = 9 ($P < 0.00001$); $I^2 = 95\%$
Test for overall effect: Z = 1.56 (P = 0.12)
Test for subgroup differences: $Chi^2 = 3.91$, df = 1 (P = 0.05), $I^2 = 74.4\%$

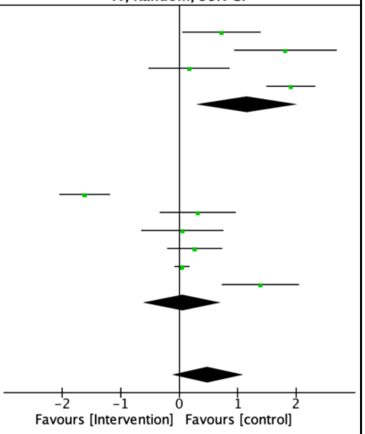

1.3. Subgroup analysis of the < 6 months group and the ≥ 6 months group

Study or Subgroup	Intervention Mean	Intervention SD	Intervention Total	Control Mean	Control SD	Control Total	Weight	Std. Mean Difference IV, Random, 95% CI
**3.1.1 <6 momths**								
Ko et al., 2020	50.7	4.9	20	45.3	9.3	18	9.8%	0.72 [0.06, 1.38]
Maitre et al., 2020	11.7	5.7	37	10.1	6.3	36	10.4%	0.26 [−0.20, 0.72]
Mann et al., 2012	14.33	1.02	15	12.43	1.02	15	9.0%	1.81 [0.94, 2.68]
Nissim et al., 2024	156.66	40	18	149.4	45.12	15	9.7%	0.17 [−0.52, 0.85]
**Subtotal (95% CI)**			**90**			**84**	**38.8%**	**0.68 [0.05, 1.30]**
Heterogeneity: $Tau^2 = 0.29$; $Chi^2 = 11.05$, df = 3 (P = 0.01); $I^2 = 73\%$								
Test for overall effect: Z = 2.13 (P = 0.03)								
**3.1.2 ≥6 months**								
Alberge et al., 2023	91.4	1.3	57	93.6	1.4	57	10.5%	−1.62 [−2.04, −1.19]
Hielkema et al., 2019	81.75	5.82	23	80	4.66	16	9.8%	0.32 [−0.32, 0.96]
Kara et al., 2019	4	2.06	16	3.87	2.65	16	9.6%	0.05 [−0.64, 0.75]
Manaseki-Holland et al., 2010	100.34	14.31	536	99.64	15.79	564	11.0%	0.05 [−0.07, 0.16]
Van Hus et al., 2016	0.12	0.12	69	−0.12	0.13	67	10.5%	1.91 [1.50, 2.32]
Yildiz et al., 2025	107.82	11.78	23	89.52	14.05	23	9.8%	1.39 [0.74, 2.04]
**Subtotal (95% CI)**			**724**			**743**	**61.2%**	**0.34 [−0.56, 1.25]**
Heterogeneity: $Tau^2 = 1.21$; $Chi^2 = 154.81$, df = 5 (P < 0.00001); $I^2 = 97\%$								
Test for overall effect: Z = 0.74 (P = 0.46)								
**Total (95% CI)**			**814**			**827**	**100.0%**	**0.49 [−0.12, 1.10]**
Heterogeneity: $Tau^2 = 0.88$; $Chi^2 = 172.52$, df = 9 (P < 0.00001); $I^2 = 95\%$								
Test for overall effect: Z = 1.56 (P = 0.12)								
Test for subgroup differences: $Chi^2 = 0.36$, df = 1 (P = 0.55), $I^2 = 0\%$								

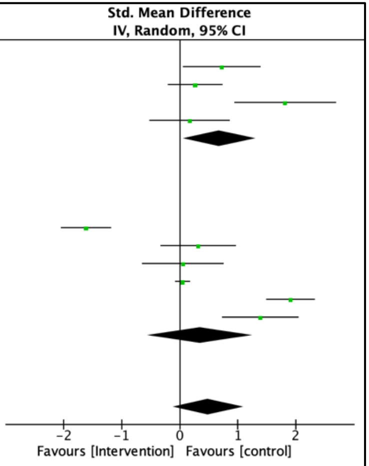

Figure S2. The effects of early interventions on cognitive development

Study or Subgroup	Intervention Mean	Intervention SD	Intervention Total	Control Mean	Control SD	Control Total	Weight	Std. Mean Difference IV, Random, 95% CI
Alberge et al., 2023	91.6	1.3	57	93.1	1.3	57	20.5%	−1.15 [−1.54, −0.75]
Ko et al., 2020	137.8	14.4	20	128.6	16.8	18	19.6%	0.58 [−0.07, 1.23]
Landa et al., 2011	36.75	14.54	24	32.24	14.07	24	19.9%	0.31 [−0.26, 0.88]
Van Hus et al., 2016	0.07	0.13	69	−0.07	0.13	67	20.6%	1.07 [0.71, 1.43]
Yildiz et al., 2025	115	10.22	23	94.56	10.54	23	19.3%	1.94 [1.22, 2.65]
**Total (95% CI)**			**193**			**189**	**100.0%**	**0.53 [−0.53, 1.60]**

Heterogeneity: $Tau^2 = 1.40$; $Chi^2 = 89.49$, df = 4 (P < 0.00001); $I^2 = 96\%$
Test for overall effect: Z = 0.98 (P = 0.33)

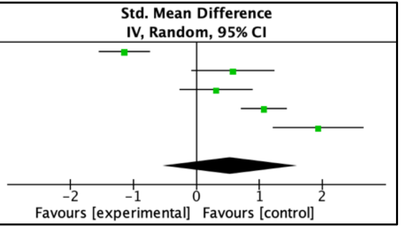

2.1. Subgroup analysis of the mixed group and the preterm group

Study or Subgroup	Intervention Mean	Intervention SD	Intervention Total	Control Mean	Control SD	Control Total	Weight	Std. Mean Difference IV, Random, 95% CI
**1.2.1 Mixed**								
Ko et al., 2020	137.8	14.4	20	128.6	16.8	18	19.6%	0.58 [−0.07, 1.23]
Landa et al., 2011	36.75	14.54	24	32.24	14.07	24	19.9%	0.31 [−0.26, 0.88]
Yildiz et al., 2025	115	10.22	23	94.56	10.54	23	19.3%	1.94 [1.22, 2.65]
**Subtotal (95% CI)**			**67**			**65**	**58.8%**	**0.92 [−0.02, 1.87]**
Heterogeneity: $Tau^2 = 0.59$; $Chi^2 = 13.07$, df = 2 (P = 0.001); $I^2 = 85\%$								
Test for overall effect: Z = 1.91 (P = 0.06)								
**1.2.2 Preterm**								
Alberge et al., 2023	91.6	1.3	57	93.1	1.3	57	20.5%	−1.15 [−1.54, −0.75]
Van Hus et al., 2016	0.07	0.13	69	−0.07	0.13	67	20.6%	1.07 [0.71, 1.43]
**Subtotal (95% CI)**			**126**			**124**	**41.2%**	**−0.04 [−2.21, 2.14]**
Heterogeneity: $Tau^2 = 2.42$; $Chi^2 = 65.70$, df = 1 (P < 0.00001); $I^2 = 98\%$								
Test for overall effect: Z = 0.03 (P = 0.97)								
**Total (95% CI)**			**193**			**189**	**100.0%**	**0.53 [−0.53, 1.60]**

Heterogeneity: $Tau^2 = 1.40$; $Chi^2 = 89.49$, df = 4 (P < 0.00001); $I^2 = 96\%$
Test for overall effect: Z = 0.98 (P = 0.33)
Test for subgroup differences: $Chi^2 = 0.63$, df = 1 (P = 0.43), $I^2 = 0\%$

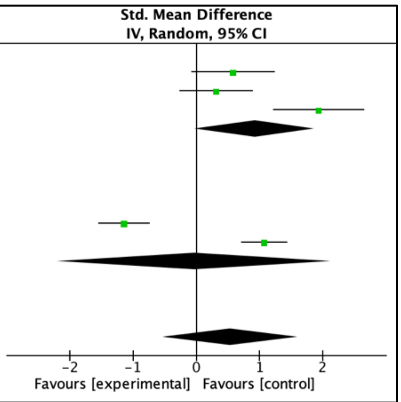

2.2. Subgroup analysis of the professional group and the home group

Study or Subgroup	Intervention Mean	Intervention SD	Intervention Total	Control Mean	Control SD	Control Total	Weight	Std. Mean Difference IV, Random, 95% CI
**2.2.1 Professional**								
Ko et al., 2020	137.8	14.4	20	128.6	16.8	18	19.6%	0.58 [−0.07, 1.23]
Landa et al., 2011	36.75	14.54	24	32.24	14.07	24	19.9%	0.31 [−0.26, 0.88]
Van Hus et al., 2016	0.07	0.13	69	−0.07	0.13	67	20.6%	1.07 [0.71, 1.43]
**Subtotal (95% CI)**			**113**			**109**	**60.2%**	**0.70 [0.20, 1.19]**
Heterogeneity: $Tau^2 = 0.12$; $Chi^2 = 5.46$, df = 2 (P = 0.07); $I^2 = 63\%$								
Test for overall effect: Z = 2.77 (P = 0.006)								
**2.2.2 Home**								
Alberge et al., 2023	91.6	1.3	57	93.1	1.3	57	20.5%	−1.15 [−1.54, −0.75]
Yildiz et al., 2025	115	10.22	23	94.56	10.54	23	19.3%	1.94 [1.22, 2.65]
**Subtotal (95% CI)**			**80**			**80**	**39.8%**	**0.38 [−2.64, 3.40]**
Heterogeneity: $Tau^2 = 4.66$; $Chi^2 = 55.03$, df = 1 (P < 0.00001); $I^2 = 98\%$								
Test for overall effect: Z = 0.25 (P = 0.81)								
**Total (95% CI)**			**193**			**189**	**100.0%**	**0.53 [−0.53, 1.60]**

Heterogeneity: $Tau^2 = 1.40$; $Chi^2 = 89.49$, df = 4 (P < 0.00001); $I^2 = 96\%$
Test for overall effect: Z = 0.98 (P = 0.33)
Test for subgroup differences: $Chi^2 = 0.04$, df = 1 (P = 0.84), $I^2 = 0\%$

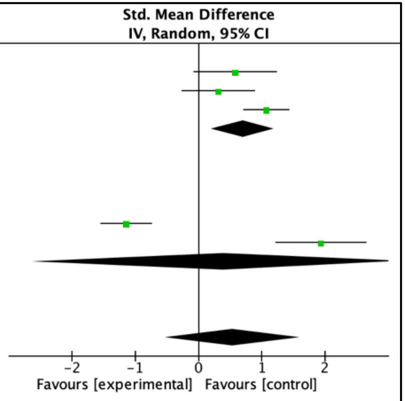

2.3. Subgroup analysis of the < 6 months group and the ≥ 6 months group

Study or Subgroup	Intervention Mean	Intervention SD	Intervention Total	Control Mean	Control SD	Control Total	Weight	Std. Mean Difference IV, Random, 95% CI
**3.2.1 <6 momths**								
Ko et al., 2020	137.8	14.4	20	128.6	16.8	18	19.6%	0.58 [−0.07, 1.23]
Yildiz et al., 2025	115	10.22	23	94.56	10.54	23	19.3%	1.94 [1.22, 2.65]
**Subtotal (95% CI)**			**43**			**41**	**38.9%**	**1.25 [−0.08, 2.58]**
Heterogeneity: $Tau^2 = 0.80$; $Chi^2 = 7.61$, df = 1 (P = 0.006); $I^2 = 87\%$								
Test for overall effect: Z = 1.84 (P = 0.07)								
**3.2.2 ≥6 months**								
Alberge et al., 2023	91.6	1.3	57	93.1	1.3	57	20.5%	−1.15 [−1.54, −0.75]
Landa et al., 2011	36.75	14.54	24	32.24	14.07	24	19.9%	0.31 [−0.26, 0.88]
Van Hus et al., 2016	0.07	0.13	69	−0.07	0.13	67	20.6%	1.07 [0.71, 1.43]
**Subtotal (95% CI)**			**150**			**148**	**61.1%**	**0.08 [−1.36, 1.51]**
Heterogeneity: $Tau^2 = 1.55$; $Chi^2 = 66.25$, df = 2 (P < 0.00001); $I^2 = 97\%$								
Test for overall effect: Z = 0.11 (P = 0.92)								
**Total (95% CI)**			**193**			**189**	**100.0%**	**0.53 [−0.53, 1.60]**

Heterogeneity: $Tau^2 = 1.40$; $Chi^2 = 89.49$, df = 4 (P < 0.00001); $I^2 = 96\%$
Test for overall effect: Z = 0.98 (P = 0.33)
Test for subgroup differences: $Chi^2 = 1.38$, df = 1 (P = 0.24), $I^2 = 27.4\%$

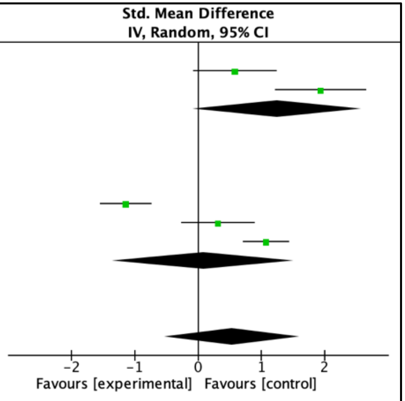

Figure S3. The effects of early interventions on language development

Study or Subgroup	Intervention Mean	SD	Total	Control Mean	SD	Total	Weight	Std. Mean Difference IV, Random, 95% CI
Alberge et al., 2023	88.7	1.6	57	88.7	1.7	57	29.2%	0.00 [−0.37, 0.37]
Kaiser et al., 2017	473.9	213.8	8	432.9	263.6	11	20.3%	0.16 [−0.75, 1.07]
Landa et al., 2011	34.08	14.59	24	31.92	13.67	24	26.1%	0.15 [−0.42, 0.72]
Yildiz et al., 2025	124.39	16.35	23	103.86	9.18	23	24.5%	1.52 [0.86, 2.19]
**Total (95% CI)**			**112**			**115**	**100.0%**	**0.44 [−0.24, 1.12]**

Heterogeneity: Tau² = 0.38; Chi² = 15.96, df = 3 (P = 0.001); I² = 81%
Test for overall effect: Z = 1.28 (P = 0.20)

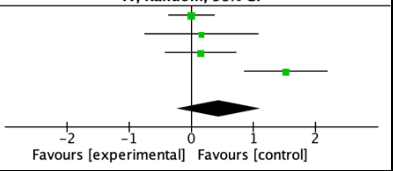

3.1. Subgroup analysis of the mixed group and the preterm group

Study or Subgroup	Intervention Mean	SD	Total	Control Mean	SD	Total	Weight	Std. Mean Difference IV, Random, 95% CI
**1.3.1 Mixed**								
Kaiser et al., 2017	473.9	213.8	8	432.9	263.6	11	20.3%	0.16 [−0.75, 1.07]
Landa et al., 2011	34.08	14.59	24	31.92	13.67	24	26.1%	0.15 [−0.42, 0.72]
Yildiz et al., 2025	124.39	16.35	23	103.86	9.18	23	24.5%	1.52 [0.86, 2.19]
**Subtotal (95% CI)**			**55**			**58**	**70.8%**	**0.62 [−0.31, 1.56]**
Heterogeneity: Tau² = 0.55; Chi² = 10.72, df = 2 (P = 0.005); I² = 81%								
Test for overall effect: Z = 1.30 (P = 0.19)								
**1.3.2 Preterm**								
Alberge et al., 2023	88.7	1.6	57	88.7	1.7	57	29.2%	0.00 [−0.37, 0.37]
**Subtotal (95% CI)**			**57**			**57**	**29.2%**	**0.00 [−0.37, 0.37]**
Heterogeneity: Not applicable								
Test for overall effect: Z = 0.00 (P = 1.00)								
**Total (95% CI)**			**112**			**115**	**100.0%**	**0.44 [−0.24, 1.12]**

Heterogeneity: Tau² = 0.38; Chi² = 15.96, df = 3 (P = 0.001); I² = 81%
Test for overall effect: Z = 1.28 (P = 0.20)
Test for subgroup differences: Chi² = 1.48, df = 1 (P = 0.22), I² = 32.2%

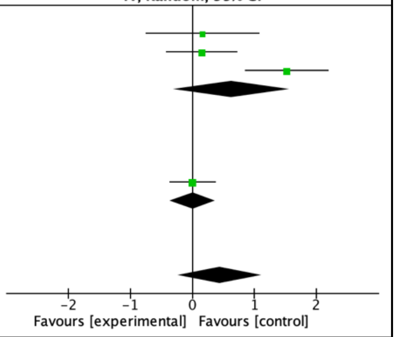

3.2. Subgroup analysis of the professional group and the home group

Study or Subgroup	Intervention Mean	SD	Total	Control Mean	SD	Total	Weight	Std. Mean Difference IV, Random, 95% CI
**2.3.1 Professional**								
Kaiser et al., 2017	473.9	213.8	8	432.9	263.6	11	20.3%	0.16 [−0.75, 1.07]
Landa et al., 2011	34.08	14.59	24	31.92	13.67	24	26.1%	0.15 [−0.42, 0.72]
**Subtotal (95% CI)**			**32**			**35**	**46.4%**	**0.15 [−0.33, 0.63]**
Heterogeneity: Tau² = 0.00; Chi² = 0.00, df = 1 (P = 0.99); I² = 0%								
Test for overall effect: Z = 0.62 (P = 0.53)								
**2.3.2 Home**								
Alberge et al., 2023	88.7	1.6	57	88.7	1.7	57	29.2%	0.00 [−0.37, 0.37]
Yildiz et al., 2025	124.39	16.35	23	103.86	9.18	23	24.5%	1.52 [0.86, 2.19]
**Subtotal (95% CI)**			**80**			**80**	**53.6%**	**0.73 [−0.76, 2.23]**
Heterogeneity: Tau² = 1.08; Chi² = 15.48, df = 1 (P < 0.0001); I² = 94%								
Test for overall effect: Z = 0.97 (P = 0.33)								
**Total (95% CI)**			**112**			**115**	**100.0%**	**0.44 [−0.24, 1.12]**

Heterogeneity: Tau² = 0.38; Chi² = 15.96, df = 3 (P = 0.001); I² = 81%
Test for overall effect: Z = 1.28 (P = 0.20)
Test for subgroup differences: Chi² = 0.53, df = 1 (P = 0.47), I² = 0%

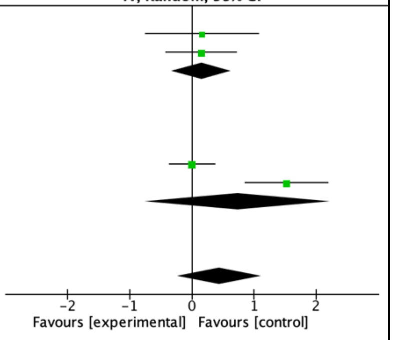

3.3. Subgroup analysis of the < 6 months group and the ≥ 6 months group

Study or Subgroup	Intervention Mean	SD	Total	Control Mean	SD	Total	Weight	Std. Mean Difference IV, Random, 95% CI
**3.3.1 <6 momths**								
Yildiz et al., 2025	124.39	16.35	23	103.86	9.18	23	24.5%	1.52 [0.86, 2.19]
**Subtotal (95% CI)**			**23**			**23**	**24.5%**	**1.52 [0.86, 2.19]**
Heterogeneity: Not applicable								
Test for overall effect: Z = 4.50 (P < 0.00001)								
**3.3.2 ≥6 months**								
Alberge et al., 2023	88.7	1.6	57	88.7	1.7	57	29.2%	0.00 [−0.37, 0.37]
Kaiser et al., 2017	473.9	213.8	8	432.9	263.6	11	20.3%	0.16 [−0.75, 1.07]
Landa et al., 2011	34.08	14.59	24	31.92	13.67	24	26.1%	0.15 [−0.42, 0.72]
**Subtotal (95% CI)**			**89**			**92**	**75.5%**	**0.06 [−0.24, 0.35]**
Heterogeneity: Tau² = 0.00; Chi² = 0.25, df = 2 (P = 0.88); I² = 0%								
Test for overall effect: Z = 0.38 (P = 0.71)								
**Total (95% CI)**			**112**			**115**	**100.0%**	**0.44 [−0.24, 1.12]**

Heterogeneity: Tau² = 0.38; Chi² = 15.96, df = 3 (P = 0.001); I² = 81%
Test for overall effect: Z = 1.28 (P = 0.20)
Test for subgroup differences: Chi² = 15.72, df = 1 (P < 0.0001), I² = 93.6%

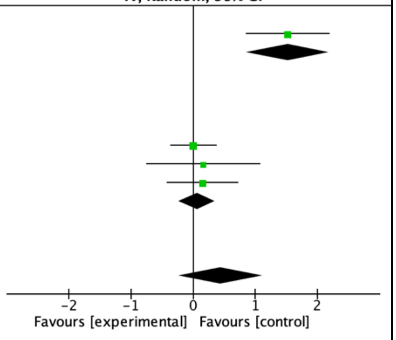

Figure S4. The effects of early interventions on physical growth

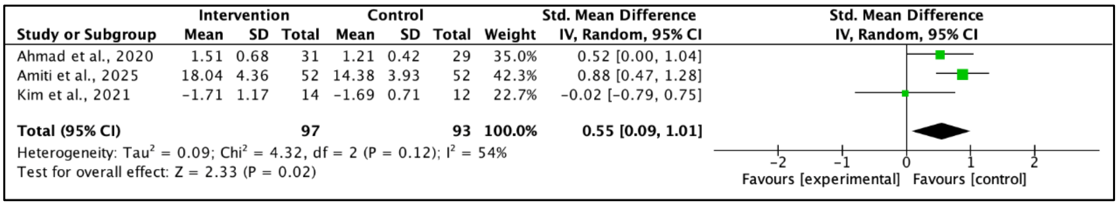

4.1. Subgroup analysis of the mixed group and the preterm group

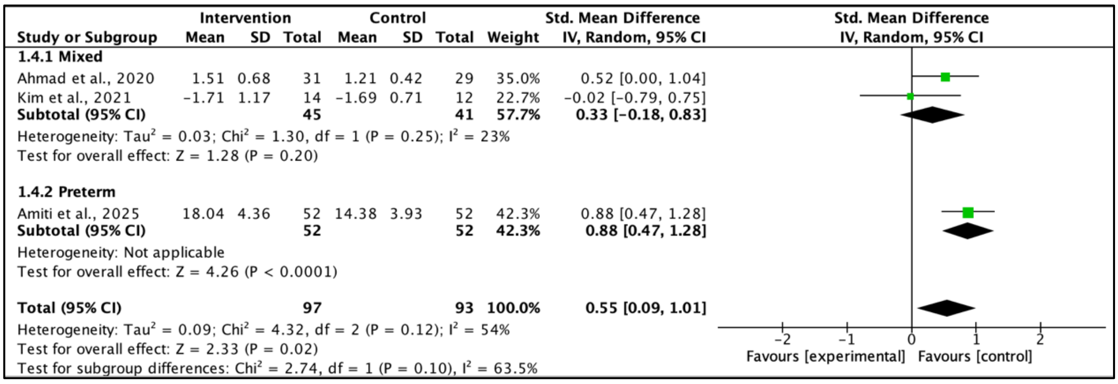

4.2. Subgroup analysis of the professional group and the home group

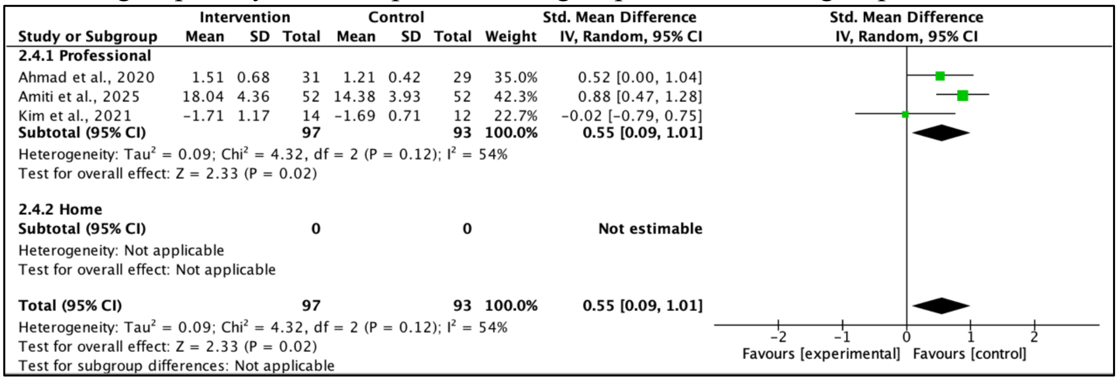

4.3. Subgroup analysis of the < 6 months group and the ≥ 6 months group

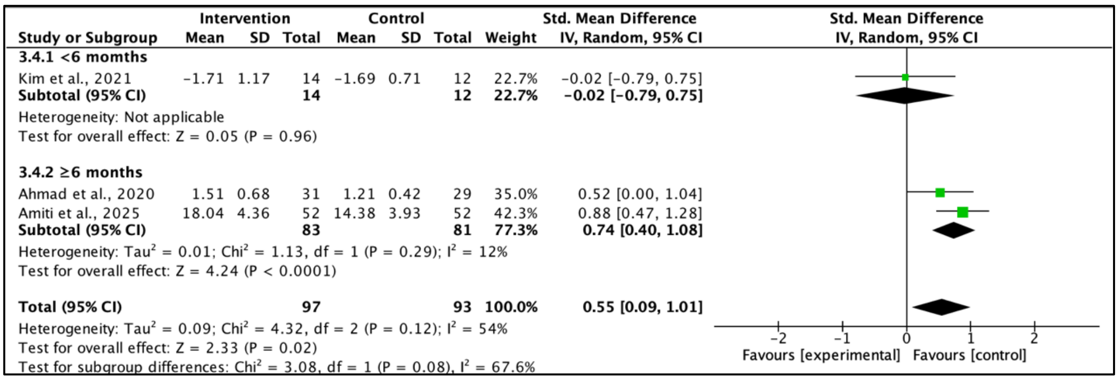

Figure S5. The funnel plot for motor outcomes

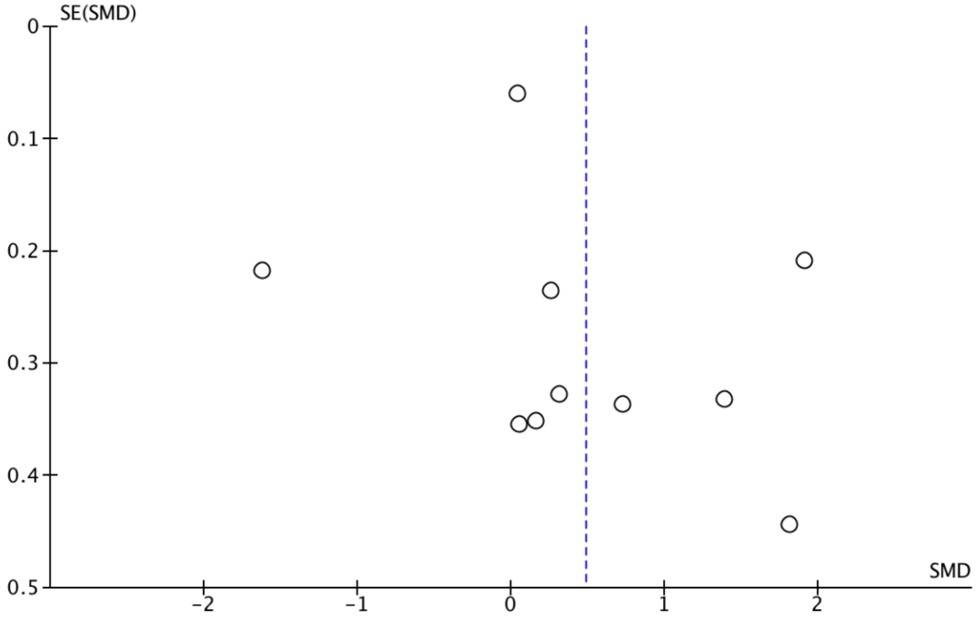

Figure S6. The Egger’s regression plot

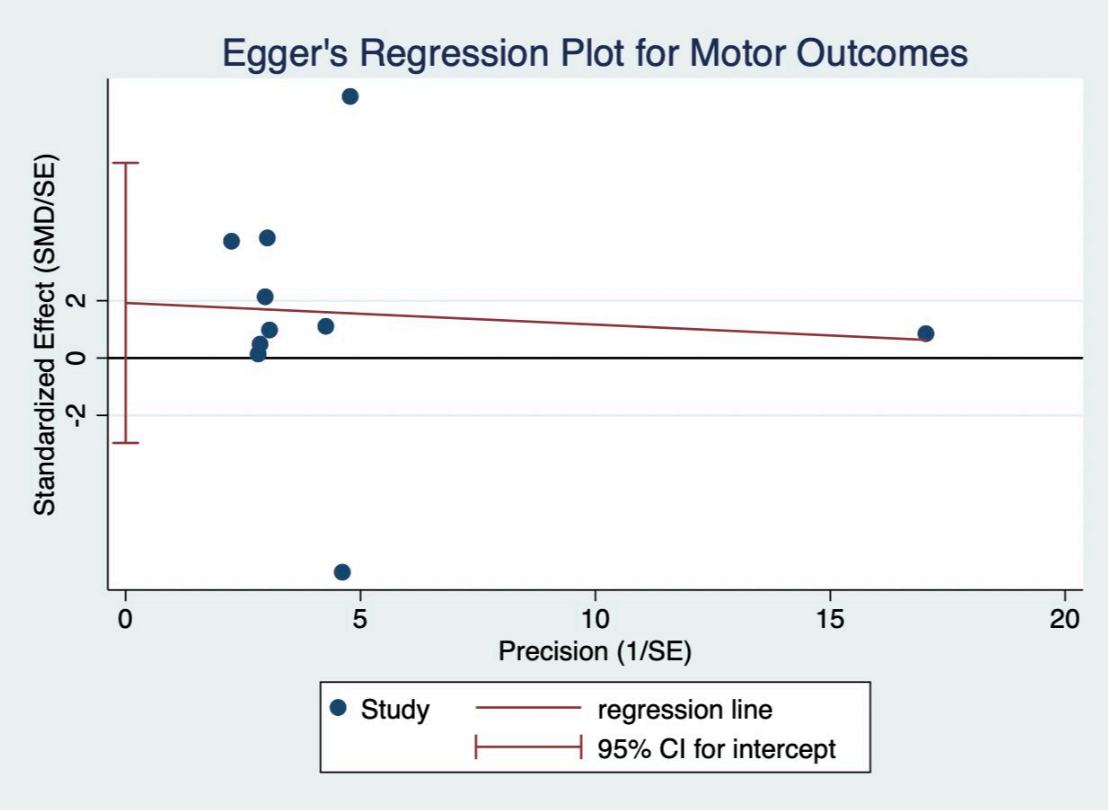

Table S1. A summary of findings from the certainty assessment

Certainty assessment							No. of patients		Effect		Certainty	Importance
No. of studies	Study design	Risk of bias	Inconsistency	Indirectness	Imprecision	Other considerations	Early intervention	Routine care	Relative (95% CI)	Absolute (95% CI)		
Motor function												
10	randomised trials	not serious	serious[a]	not serious	serious[b]	none	814	827	-	SMD 0.49 higher (0.12 lower to 1.1 higher)	⊕⊕◯◯ Low[a,b]	CRITICAL
Cognitive function												
5	randomised trials	serious[c]	serious[a]	not serious	serious[b]	none	193	189	-	SMD 0.53 higher (0.53 lower to 1.6 higher)	⊕◯◯◯ Very low[a,b,c]	CRITICAL
Language function												
4	randomised trials	serious[d]	serious[a]	not serious	serious[b]	none	112	115	-	SMD 0.44 higher (0.24 lower to 1.12 higher)	⊕◯◯◯ Very low[a,b,d]	CRITICAL
Physical growth												
3	randomised trials	not serious	not serious	not serious	serious[b]	none	97	93	-	SMD 0.55 higher (0.09 higher to 1.01 higher)	⊕⊕⊕◯ Moderate[b]	CRITICAL

a. Downgraded by one level: high statistical heterogeneity.

b. Downgraded by one level: the 95% CI was wide and crossed the line of no effect.

c. Downgraded by one level: the five studies included four "some concerns" and one "low risk".

d. Downgraded by one level: the four studies included three "some concerns" and one "high risk".

Table S2. Leave-one-out sensitivity analysis for overall meta-analyses

Excluded study	Pooled SMD (95% CI)	I^2	τ^2
**Motor function**			
Alberge et al., 2023	0.73 [0.18, 1.28]	92%	0.61
Hielkema et al., 2019	0.51 [-0.16, 1.17]	95%	0.96
Kara et al., 2019	0.53 [-0.13, 1.20]	95%	0.95
Ko et al., 2020	0.46 [-0.20, 1.12]	95%	0.94
Maitre et al., 2020	0.51 [-0.18, 1.21]	95%	1.04
Manaseki-Holland et al., 2010	0.55 [-0.31, 1.40]	95%	1.60
Mann et al., 2012	0.35 [-0.27, 0.98]	95%	0.85
Nissim et al., 2024	0.52 [-0.14, 1.19]	95%	0.95
Van Hus et al., 2016	0.31 [-0.23, 0.85]	92%	0.59
Yildiz et al., 2025	0.39 [-0.25, 1.03]	95%	0.87
**Cognitive function**			
Alberge et al., 2023	0.96 [0.36, 1.55]	78%	0.29
Ko et al., 2020	0.53 [-0.78, 1.83]	97%	1.69
Landa et al., 2011	0.59 [-0.75, 1.94]	97%	1.81
Van Hus et al., 2016	0.40 [-0.91, 1.71]	95%	1.69
Yildiz et al., 2025	0.40 [-0.91, 1.71]	96%	1.24
**Language function**			
Alberge et al., 2023	0.62 [-0.31, 1.56]	81%	0.55
Kaiser et al., 2017	0.52 [-0.33, 1.38]	87%	0.49
Landa et al., 2011	0.56 [-0.45, 1.56]	87%	0.68
Yildiz et al., 2025	0.06 [-0.24, 0.35]	0%	0.00
**Physical growth**			
Ahmad et al., 2020	0.49 [-0.38, 1.36]	75%	0.30
Amiti et al., 2025	0.33 [-0.18, 0.83]	23%	0.03
Kim et al., 2021	0.74 [0.40, 1.08]	12%	0.01
